# An examination of the influence of prefrontal cortical brain stimulation on sexual decision making

**DOI:** 10.1093/oons/kvag003

**Published:** 2026-05-15

**Authors:** Michael T Shaw, Leah Cingranelli, Simona Kobryn, Isabella Tavarez, Alberto Torres-Aragón, Christina M Balderrama-Durbin, Brandon E Gibb, Leigh E Charvet, Richard E Mattson

**Affiliations:** Department of Psychology, Binghamton University, 4400 Vestal Parkway East, Binghamton, NY, 13902-6000, United States; Department of Psychology, Binghamton University, 4400 Vestal Parkway East, Binghamton, NY, 13902-6000, United States; Department of Psychology, University of Maine, College Avenue, Orono, ME, 04473, United States; Department of Psychology, Binghamton University, 4400 Vestal Parkway East, Binghamton, NY, 13902-6000, United States; Department of Psychology, Binghamton University, 4400 Vestal Parkway East, Binghamton, NY, 13902-6000, United States; Department of Psychology, Binghamton University, 4400 Vestal Parkway East, Binghamton, NY, 13902-6000, United States; Department of Psychology, Binghamton University, 4400 Vestal Parkway East, Binghamton, NY, 13902-6000, United States; Department of Psychology, Binghamton University, 4400 Vestal Parkway East, Binghamton, NY, 13902-6000, United States; Department of Neurology, New York University Grossman School of Medicine, 550 1st Avenue, New York, NY, 10016, United States; Department of Psychology, Binghamton University, 4400 Vestal Parkway East, Binghamton, NY, 13902-6000, United States

**Keywords:** dating violence, sexual aggression, decision making, neuroscience, transcranial direct current stimulation

## Abstract

The goal of this study was to clarify the contribution of prefrontal cortex activity to men’s appraisals, affect states, and decision making in the context of hypothetical heterosexual dating scenarios lacking clear consent. Transcranial direct stimulation (tDCS) was applied to the prefrontal cortex to examine whether it would modify and reduce the likelihood of hypothetical dating violence. Participants (*n* = 102) responded to first-person vignettes wherein a woman responded either ambiguously or with a refusal to their sexual advance. While responding to the vignettes, participants received sham or active tDCS to modulate prefrontal cortex-mediated responding. Neuromodulation interacted with the communication response type of the woman in the vignette (e.g. ambiguous vs. refusal responses) to influence participants’ reports of negative affect (*F* = 9.57, *P* = 0.002, Cohen’s *f^2^* = 0.02) and their sexual decision making (*F* = 5.06, *P* = 0.025, Cohen’s *f^2^* = 0.003). For example, participants that received a refusal from a woman while receiving tDCS were more likely to report greater negative affect and a reduced likelihood of sexual behavior as compared with vignettes including an ambiguous response. These findings highlight the complex interplay of situational context on affect and social behavior while providing initial support for the utility of brain stimulation techniques in developing a mechanistic, neurocognitive understanding of the prefrontal cortex’s role in men's sexual decision making in dating scenarios.

## Introduction

The effects of sexual aggression are significant and carry serious mental health consequences (Campbell et al. 2009, Dworkin et al. 2021). Women experience greater rates of sexual aggression by men (See: Dartnall and Jewkes 2013, Jewkes et al. 2013), particularly in the context of dating (Hines et al. 2012). A potential mechanism of men’s sexual aggression in dating violence are their cognitive appraisals within sexual situations. Men notably overestimate the sexual intentions of women (Farris et al. 2008), appraise women’s non-sexual behaviors (e.g. smiling) in a sexualized manner (Abbey et al. 2011, Treat and Viken 2018), and place greater stock on non-verbal cues or situational context (e.g. women’s alcohol use) that do not affirmatively signal consent to sexual activity (King et al. 2021, Mattson et al. 2022). Recent evidence has also demonstrated that men at-risk to engage in dating violence may appraise sexual consent or willingness in sexually ambiguous scenarios (e.g. passive female responding; Anduze et al. 2024) using problematic schemas related to consent perception (e.g. rape myths), which are linked to sexual advances in the face of overt refusals (McKinnon et al. 2025).

Appraisals of sexual consent reflect a cognitive and social process whereby individuals attend to communication and situational cues, decode their social significance, and draw inferences regarding a person’s internal level of willingness or consent, which then informs their behavior (cf. Lemerise and Arsenio 2000). Much of this cognitive processing is accomplished via the functional activity of the prefrontal cortex (PFC; Friedman and Robbins 2022) – a critical structure for appraising information and exerting self-control (Gläscher et al. 2012). There is also evidence that the PFC influences sexual behavior (Spinella 2007). This has perhaps been most apparent among investigations with sex-offending samples, which have highlighted atypical PFC functioning and related cognitive deficits in this population (Kirk-Provencher et al. 2020). Other lines of inquiry have examined how the PFC’s regulatory influence on sexual decision making can be temporarily diminished (Salehinejad et al. 2021). For example, sexual arousal inductions have been demonstrated to override cognitive control and support men’s rape proclivity (Craig et al. 2022). Additionally, alcohol use, which disrupts PFC functioning, has been shown to facilitate hypothetical dating violence via state changes in anger (Davis et al. 2021) and executive control (Gallagher et al. 2010). Despite these converging lines of evidence, basic psychological science has yet to examine how PFC functioning informs appraisals of sexual consent and decision making in the context of dating violence.

To date, the study of dating violence has only sparingly integrated neuroscience perspectives (e.g. Mbiydzenyuy et al. 2024, Toates et al. 2017, Victor and Hariri 2016). Indeed, the dating violence literature has largely been constrained to surveys, observational research (McDermott et al. 2015), and experiments that manipulate information associated with hypothetical sexual encounters (Treat et al. 2016) or administer substances to participants such as alcohol (e.g. Abbey 2011). Although these investigations have been useful in characterizing individuals that are more likely to engage in sexual assault, as well as the relevant contexts and circumstances for these behaviors (Tharp et al. 2013), they have only revealed glimpses into the neurocognitive mechanisms that enable dating violence (Davis et al. 2018).

The current work examined how PFC activity is associated with hypothetical sexual appraisals and decision making with the use of a non-invasive brain stimulation methodology. Transcranial direct current stimulation (tDCS) is a safe and well-studied experimental manipulation that can modify the membrane potential of neurons via the application of a low-intensity electrical current to the scalp (Brunoni et al. 2012) and has been found to influence cognitive processing when directed at the PFC (Kuo and Nitsche 2012). In the current study, we used tDCS to assess how an electrical field induced in this region would impact men’s perceptions and behavior in simulated dating encounters. More specifically, heterosexual young men were recruited and randomized to receive either active or sham tDCS while responding to a series of literary vignettes that outlined a hypothetical heterosexual ‘hookup.’ The hookup scenarios varied factorially, with multiple situational elements differing between trials (e.g. the level of sexual intimacy and women’s communication; see Lofgreen et al. 2021). Vignette scenarios were ambiguous in that they did not specify any obvious messages of sexual willingness from women, with the goal of examining how men would appraise consent differently under tDCS stimulation, and how they might feel or behave in heteronormative situations where women’s consent is uncertain (Anduze et al. 2024).

It was hypothesized that individuals who received active tDCS, as compared with those in the sham condition, would report more conservative appraisals of consent and attenuated affective reactions, and a reduced likelihood of initiating sexual behavior. This was predicated on basic neuromodulation principles (Luan et al. 2014) and prior research suggesting that the prefrontal application of tDCS can regulate social behavior (Bell and DeWall 2018). For example, tDCS has been demonstrated to influence moral judgments of hypothetical scenarios, risk-taking, and decision making (Choi et al. 2016). Finally, exploratory analyses tested whether tDCS interacted with vignette characteristics (e.g. descriptions of alcohol use, sexual intimacy, and a fictional woman’s communication) to impact study outcomes, based on evidence that the effects of tDCS are state-dependent (Esmaeilpour et al. 2018). However, no specific predictions were made regarding such interactions given the lack of prior research in this area.

## Materials and methods

### Participants and procedure

Members of the public identifying as heterosexual men between the ages of 18–24 were screened and recruited to evaluate the aims of this experimental study. The purpose of this recruitment strategy was to sample the young adult, college-aged male population that has previously been described as being more likely to engage in dating violence (Spencer et al. 2022). Aside from the requirement that participants be fluent in the English language, other eligibility criteria that are typically used in the neuromodulation field were specified, including: Any individuals with a history of epilepsy or seizure disorder, uncontrolled migraines in the previous six months, a history of head trauma or medical implants in the head or neck, skin disorders or sensitive skin near the electrode sites (e.g. the forehead and hairline), treatment for a communicable skin infection over the previous twelve months, a history of clinically significant abnormalities on an electrocardiogram, or individuals with a pacemaker were ineligible to participate in the current study.

All study procedures were approved by the Binghamton University Institutional Review Board and the study was conducted in accordance with the Declaration of Helsinki. Individuals were compensated $30 for their participation. Participant recruitment began February 2023 and concluded in March 2024. Participants first consented to an experimental protocol using an online Qualtrics form followed by a self-report screening to determine their eligibility to participate in the current study. If eligible, participants completed an online study visit as a baseline assessment of their sexual decision making with the use of dating vignettes. Participants were then scheduled to attend an in-person visit to complete a second set of vignette outcome measures while receiving tDCS at least two days following the online survey to limit carry-over effects (*M* = 13.14 days, *SD* = 11.83 days, range: 2–66 days^1^).

A total of 110 participants were recruited via email listservs, public and bus fliers, and social media advertisements. This sample completed various self-report psychological measures with a low level of missingness that was determined to be missing completely at random (*χ^2^* = 4179.33, *df* = 6945, *P* = 0.999). Additionally, three individuals were identified as statistical multivariate outliers using Mahalanobis distances and excluded.

After excluded outliers, the final sample consisted of 102 young adult men (*M* = 20.04, *SD* = 1.75). The sample largely included individuals attending college or university (96.10%). Most of these individuals identified as White (56.90%), with smaller subsets of individuals identifying as Asian (19.60%), as more than one race (13.70%), Black (5.90%), ‘other’ (2.90%), or Hispanic (1.00%). The sample varied in their sexual histories and relationship statuses. Sixty-one percent of the sample (*n* = 62) reported prior sexual intercourse with a woman and all identified as heterosexual males at screening with no history of sexual interaction with other men. One participant changed their reported sexual orientation from heterosexual at screening to queer at follow-up, however this individual reported no prior history of sexual intercourse with a man. Sensitivity analyses demonstrated that their inclusion in analyses did not alter the pattern of findings and they were presently included in the analyzed sample. Individuals who reported previous sexual intercourse with women reported a median number of 1.50 lifetime sexual partners (range = 1–12). Additionally, more than half of the sample reported their relationship status as single (61.80%), with some individuals in self-described ‘serious’ relationships (29.40%) and a smaller group in ‘casual’ relationships (8.80%).

Participants were randomly assigned to one of two study conditions, the active tDCS or sham groups. Participants initially completed a minute of stimulation at 2.0 mA to confirm that they could tolerate the experimental procedures. Largely, stimulation was found to be tolerable for individuals in the active condition, as most participants ultimately received the targeted 2 mA stimulation intensity (*n* = 38). Individuals that found 2.0 mA stimulation to be uncomfortable (e.g. reporting painful skin irritation or itching) were given the option to complete stimulation at lower intensities. This arrangement led to six participants receiving 1.5 mA and six receiving 1.0 mA stimulation^2^. Five individuals experienced uncomfortable skin irritation related to stimulation at any intensity and opted not to complete stimulation procedures and were excluded from analyses^3^. After these exclusions, 50 participants remained in the active stimulation group while 52 individuals remained in the sham group. At the end of the experimental procedures, an item was used to ask participants which condition they believed they received (with choices of ‘Active Stimulation’, ‘Sham Stimulation’, or ‘Prefer not to answer’). Although all participants were informed during the consent process that they might not receive active stimulation, the majority of individuals in both groups reported that they received active stimulation. Specifically, 67% of the sham group and 84% of the active group reportedly believed that they received the active condition, with no significant difference in responses between groups (χ^2^ = 4.43, *df* = 2, *P* = 0.109).

### Study outcome

This study used first-person factorial vignettes to simulate dating encounters and to determine how men would appraise them, emotionally react, and make decisions. The vignettes systematically varied story elements of a dating encounter to mimic the heterogeneity of real-world sexually encounters. Converging prior work (Anduze et al. 2024, Lofgreen et al. 2021, McKinnon et al. 2025) demonstrates that men’s varied responses to these vignettes significantly correlates with specific situational factors and traits aligned with sexual aggression, as well as historical accounts of sexual coercion and forcible rape (McKinnon et al. 2025).

Aligned with these methods (e.g. Anduze et al. 2024), six different story factors presently varied to create a narrative of a ‘hookup’ or sexual encounter following a date. Story factors were chosen due to their relation to common rape myths that some individuals stereotypically associate with consent for sexual activity. These factors were sequentially presented and included the circumstances under which the reader met the fictional woman (e.g. through a dating app vs. a bar), the level of emotional closeness between the reader the fictional date, their prior sexual history (if any), whether alcohol was used between the two during a night out, and the level of sexual behavior already engaged in during the current date. Each vignette then specified that the reader attempted to elevate the level of intimacy by beginning to engage in further sexual behavior (e.g. *‘After a few minutes of making out, the two of you are almost completely undressed. You're feeling really turned on, so you start to remove Hannah's underwear’*). After this point in the narrative, the fictional woman provided varying responses, including a refusal of the reader’s advance (e.g. ‘*In response, Hannah says 'Let's not do this right now.*’) or an ambiguous response (e.g. *‘In response, Hannah tenses up – but doesn't say anything.’*). An example of a vignette is specified in the Supplementary Material and the vignette stimuli are also available in their entirety for replication and use (see the following link for an interactive tool to download the vignettes: https://ctroir.shinyapps.io/ShinyVignettes3/).

Immediately after the participant read each vignette, they completed a series of brief self-report items regarding their appraisals of the scenario and how they would hypothetically behave. First, to assess appraisals of consent, participants rated the following via Likert scale (1 – *‘not at all’* to 7 – *‘very much’*): ‘*How much do you think your date has communicated willingness to have sex?*’ Previously, multiple iterations of this item that asked about various aspects of consent have been highly correlated (*r*s > 0.8; Anduze et al. 2024), effectively reducing to one item. On average, this sample rated the vignettes as denoting low levels of female consent for sex (*M =* 2.97, *SD* = 2.04*)*. Participants also rated the likelihood that they might feel positive/masculine affect states (e.g. ‘*excited*’ and ‘*manly*’) and negative affect states (e.g. ‘*disappointed*’) in the situation using a Likert-type scale (1 – ‘*not at all’* to 7 – ‘*extremely’*; see Supplementary Table 1). Total scores were generated by summing negative affect items (negative affect: α = 0.82, *M* = 9.27, *SD* = 4.53) and by summing together imagined positive affect states, feelings of masculinity, and sexual arousal (positive affect: α = 0.90, *M* = 17.42, *SD* = 9.20). Finally, participants reviewed a series of behavioral items and rated the likelihood that they would engage in them (1 – ‘*very unlikely’* to 7 – *‘very likely’*). The behavioral items ranged from stopping the encounter altogether to coercive and forcible behavior, but were coded subtly in text. To operationalize the likelihood of aggressive decision making, only items specifying coercive (e.g. ‘*Continue engaging in the initial activity and try to verbally convince your date to advance the activity to a more intimate level*’), transgressive, or forcible behavior were used in statistical models (See Supplementary Table 1). These items cohered together reasonably well (α = 0.78, *M* = 14.25, *SD* = 6.59), despite the variation in severity of items.

Participants completed three vignette trials with varying levels of women’s communication (one refusal and two ambiguous response vignettes) at each study visit for a total of six outcome measurements across the study. Practically, individuals that received a vignette with a verbal refusal at the first study visit would also receive a vignette with a verbal refusal at the second study visit, and so on for the ambiguous response vignettes. Despite this redundancy, all other vignette factors (e.g. the level of alcohol use) were randomly varied. Importantly, there was no clear indication of affirmative consent at any point in any vignettes. As a result, the vignettes that participants received ranged in specifying fraught situations involving sexual refusals to ambiguous situations that were uncertain and lacking in communication.

### tDCS procedure

**Figure 1:**
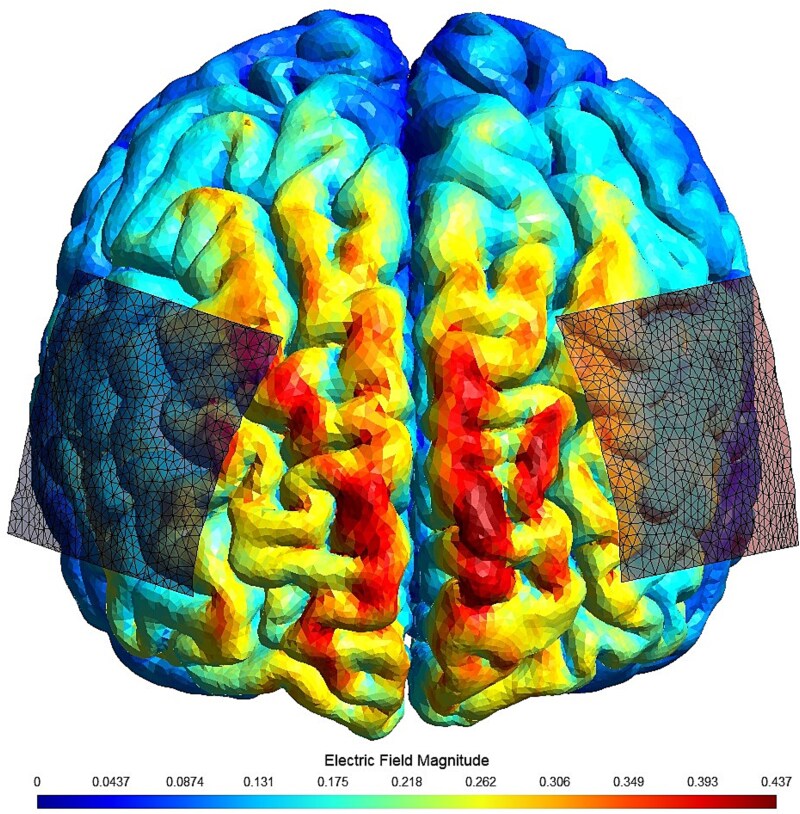
Simulated tDCS electric field. *Note.* tDCS was administered in a bilateral fashion with the anode placed over the F3 site and cathode placed over the F4 site. Sponge electrodes (5 cm by 5 cm) saturated with saline were used in the current work. The magnitude of the electric field, based on this simulation, was found to be greatest in the anterior and dorsal prefrontal regions of the brain. SIMNIBS 4.5 was used to generate this simulation (Saturnino et al. 2019).

The medical-grade Soterix 1x1 miniCT was used to stimulate the prefrontal cortex in this study. To generate excitatory stimulation of the prefrontal cortex, 5 cm by 5 cm sponge electrodes coated in saline were placed at the following sites via use of a customized head strap: Anodal electrode at F3 site and cathodal electrode at the F4 site (See Fig. 1). A stimulation intensity of 2 mA was the target stimulation intensity in the current work and most participants received 2.0 mA tDCS (*n* = 38), though a subset of participants received lower doses of stimulation for their comfort (*n* = 12). A 30-second ramp-up/ramp-down procedure was used in both the sham and active procedures. tDCS was delivered in offline and online phases. First, participants completed 10 minutes of offline tDCS. Participants were asked to sit quietly during this period and not to use their smartphones. Immediately after the ten minutes had passed, participants were instructed to respond to study vignettes while continuing to receive tDCS. Stimulation proceeded until participants completed all study outcome measures or until 20 additional minutes had passed (i.e. a maximum duration of 30 minutes of stimulation was specified). On average, participants received 26.75 (± 3.30) minutes of stimulation and the online task was found to require an average of 17.16 (± 4.46) minutes to complete.

### Analytic plan

To evaluate whether individuals’ appraisals of consent, expected affective states, and behavioral decision making changed as a function of active tDCS, a series of generalized linear mixed models (GLMM) were conducted in SPSS (Version 31). This study produced a unique nested data structure with multiple measurements of each outcome variable at both the first online session and the experimental in-person visit. To account for the nested data structure, a random intercept was estimated for each participant. Baseline vignette responses were added as covariates to GLMMs to statistically account for participants’ responding at the initial assessment. A model building approach was then utilized to evaluate the contribution of predictors sequentially. Baseline GLMMs were first conducted with only the random intercept to evaluate the variance in outcome measures between individuals. A second GLMM specified the entry of all the vignette situational characteristics. Situational characteristics that associated significantly with model outcomes were retained for a final series of GLMMs that further specified tDCS condition as a main effect, as well as its interactions with the retained situational characteristics. Due to the multiple comparisons, readers are invited to also consider the significance of results at both the 95% confidence interval (i.e. *P* < 0.05) and with a Bonferroni-corrected threshold based on the eight GLMMs conducted with fixed effects specified (i.e. *P* < 0.006).

## Results

### Appraisals of consent

The overall average of participant’s appraisals of consents (*M* = 2.97, *SD* = 2.04) revealed that the participants found the vignettes to be low in female consent or willingness for sexual behavior. However, the baseline generalized linear mixed model (AICc = 628.94) showed significant variation in consent appraisals (residuals: *b* = 0.36, *SE* = 0.04, *P* < 0.001; random intercept: *b* = 0.14, *SE* = 0.04, *P* < 0.001). Vignette factors were entered next as fixed effects in the second model (AICc = 554.18; See Supplementary Table 2 for full reporting of fixed effects tests). The response of the fictional women to the man’s advances in the vignettes was found to influence men’s appraisals, with overt refusals being associated with lower estimates of the woman’s consent compared with ambiguous responses (*F =* 8.16, *P* = 0.005, Cohen’s *f*^2^ = 0.02). Additionally, vignettes that specified more sexually intimate behavior (e.g. being undressed and engaging in oral sex) were found to positively associate with men’s estimates of women’s consent (*F* = 7.24, *P* < 0.001, Cohen’s *f*^2^ = 0.06). A final effect was found such that, if the vignette specified that the reader had used alcohol earlier in the encounter, they rated the woman’s level of consent lower (*F =* 5.77, *P* = 0.017, Cohen’s *f*^2^ = 0.01). The final model (AICc = 540.27) additionally specified tDCS condition and its interactions with the prior significant vignette factors as predictors. No main effect or interacting effect of stimulation was found on consent appraisals (*p*’s > 0.05; See Supplementary Table 3).

### Expectations of NA

**Figure 2:**
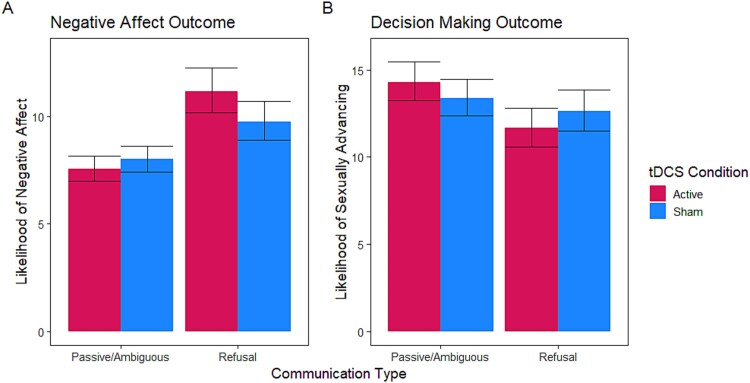
tDCS interacted with vignette details to affect Men’s responding. Caption: *Note.* Panel A. participants in both the active and sham conditions demonstrated significant within-subject changes when rating the likelihood of experiencing negative affect across communication types. Both groups rated a lower likelihood of experiencing negative affect when they imagined themselves in situations where the fictional woman responded passively or ambiguously to their advances as compared to when they received refusal responses. Additionally, participants in the active condition, relative to the sham condition, rated greater likelihood of negative affect when they received vignettes with refusal responses. Panel B. Participants in the active condition, relative to the sham condition, demonstrated significant within-subject change in their decision making across trials with different kinds of communication from the fictional woman (e.g. passive/ambiguous vs. refusal responses). Notably, this statistical finding did not survive Bonferroni correction.

The initial model (AICc = 325.06) found significant variance between individuals (random intercept: *b* = 0.06, *SE* = 0.02, *P* < 0.001) and vignettes (residuals: *b* = 0.12, *SE* = 0.01, *P* < 0.001). The subsequent model (AICc 179.22) specified situational characteristics (See Supplementary Table 4) and found that women’s communication in the vignettes influenced the readers’ imagined level of negative affect (*F* = 72.68, *P* < 0.001, Cohen’s *f*^2^ = 0.08). Women’s refusals were associated with greater likelihood of imagined negative affect. Lastly, the inclusion of the brain stimulation predictor in the final model (AICc = 130.25) demonstrated interaction effects of stimulation (*F* = 9.57, *P* = 0.002, Cohen’s *f*^2^ = 0.02; See Supplementary Table 5). Specifically, in vignettes with a refusal response, individuals who received active tDCS were more likely to experience negative affect than those who received sham tDCS (see Fig. 2, Panel A).

Post-hoc comparisons of the interaction revealed greater reports of negative affect when individuals in the active condition (mean difference = 3.62 [Refusal Condition - Passive/Ambiguous Condition], *SE* = 0.48, *t* = 7.63, *P* < 0.001) and in the sham condition (mean difference = 1.76 [Refusal Condition - Passive/Ambiguous Condition], *SE* = 0.41, *t* = 4.25, *P* < 0.001) received a refusal response, relative to a passive or ambiguous response. Additionally, individuals in the active condition who received a refusal response reported that they would experience significantly greater negative affect than those in the sham condition (mean difference = −1.40 [Sham Condition - Active Condition], *SE* = 0.69, *t* = −2.025, *P* = 0.044), but the groups did not differ significantly in their expectations of negative affect when faced with a passive or ambiguous response (*P* = 0.282).

### Expectations of PA

Individuals in the baseline positive affect model (AICc = 424.35) were found to vary significantly from each other in their reports (random intercept: *b* = 0.17, *SE* = 0.31, *P* < 0.001) and between vignettes (residuals: *b* = 0.14, *SE* = 0.01, *P* < 0.001). In the situational model (AICc = 346.03, See Supplementary Table 6 for reporting of fixed effects), vignette elements that specified that the rater ‘*felt close*’ to the woman (*F* = 5.21, *P* = 0.02, Cohen’s *f*^2^ = 0.02) or that greater levels of sexual behavior were occurring (*F* = 7.77, *P* < 0.001, Cohen’s *f*^2^ = 0.05) were associated with increased reports of male positive affect. Additionally, vignettes that specified a refusal response were associated with reduced positive affect, relative to vignettes that specified ambiguous responses to men’s advances (*F* = 32.32, *P* < 0.001, Cohen’s *f*^2^ = 0.03). No main or interacting effects of tDCS were observed in the final model (AICc = 327.19; *p*’s > 0.05; See Supplementary Table 7 for reporting of fixed effects).

### Decision making

In the decision making model series, participants’ hypothetical behavior was tested as an outcome and the sample was once more found to significantly vary between each other (random intercept: *b* = 0.10, *SE* = 0.02, *P* < 0.001) and between vignettes (residuals: *b* = 0.07, *SE* = 0.007, *P* < 0.001). Next, the situational model was conducted to examine the factors associated with men’s behavioral intentions (AICc = 182.90, See Supplementary Table 8 for reporting of fixed effects). Greater levels of sexual behavior already occurring in the vignette were positively associated with the likelihood of the man engaging in further sexual behavior (*F* = 2.72, *P* = 0.045, Cohen’s *f*^2^ = 0.02). Additionally, woman’s refusals served to reduce the likelihood of sexual behavior, relative to ambiguous responses (*F* = 13.98, *P* < 0.001, Cohen’s *f*^2^ = 0.01). A final model was conducted where tDCS condition and its interactions with other factors were specified as fixed effects (AICc = 150.67; See Supplementary Table 9). This model revealed a significant interaction of tDCS and women’s responses in the vignette (passive/ambiguous vs refusal; *F* = 5.06, *P* = 0.025, Cohen’s *f*^2^ = 0.003).

Inspection of the interaction (See Fig. 2, Panel B) revealed a similar trend as was observed for the negative affect model. Overall, the active and sham groups did not significantly differ in their sexual decision making when faced with refusals (*P* = 0.241) or passive or ambiguous responses (*P* = 0.224). However, those who received active tDCS appeared to accord their decision making more strongly to women’s communication (mean difference = −2.65 [Refusal Condition - Passive/Ambiguous Condition], *SE* = 0.59, *t* = −4.46, *P* < 0.001), whereas those in the sham condition experienced no significant difference in their responding tendency (*P* = 0.230). This pattern suggests that tDCS may have had a within-subjects effect on men’s decision making based on women’s communication. That is, active stimulation potentiated the likelihood of sexually advancing when communication was ambiguous, yet reduced the likelihood of advancement when faced with a clear refusal. However, it is important to note that this tDCS interaction effect did not survive Bonferroni correction for multiple comparisons.

## Discussion

This work contributes evidence that the PFC impacts cognitive processing during hypothetical sexual scenarios. Theoretically, PFC activity continuously modifies affective valence and behavioral selection based on moment-to-moment changes in one’s immediate environment (Domenech and Koechlin 2015). During vignette completion, brain stimulation directed at the PFC ostensibly adjusted men’s reactions to women’s refusals to result in a greater likelihood of negative, but not positive, affective responding. This significant interaction effect on hypothetical negative reactions may indicate that men in the active condition had an increased awareness that their goal, as specified at outset of the vignette (i.e. that they ‘*were hoping to have sex*’), was less likely to occur. Despite this possibility, stimulation did not alter appraisals of consent, and thus this study joins other works that have demonstrated varying effects of PFC stimulation on appraisals (e.g. Clarke et al. 2020, cf., Piretti et al. 2022). Furthermore, there is some evidence that men’s hypothetical behavioral intentions differed as a function of stimulation and interpersonal exchanges in the vignette. However, this effect did not survive Bonferroni correction for multiple comparisons and should be interpreted with caution. Future work that can explore whether and how tDCS modifies sexual decision making in other sexual contexts appears warranted.

This pattern of results may reflect successful neuromodulation of the prefrontal cortex, which consists of several cortical structures relevant to the processing of social information. We speculate here that the dorsolateral prefrontal cortex (DLPFC), which tabulates and manages situational information in individuals’ working memory (Curtis and D'Esposito 2003), may have adjusted men’s affective and behavioral responding (Ballard et al. 2011, Baumgartner et al. 2011). Based on accounts of lateral asymmetry in motivational processing (Poole and Gable 2014), the left DLPFC manages information relevant to approach and avoidance-oriented goals in working memory (Spielberg et al. 2012). In the current work, men received anodal stimulation to this cortical region while imagining a single approach-oriented goal: that they were interested in having sex. Consequently, the left DLPFC’s functional activity may have encoded for vignette information that men found to be especially relevant to the likelihood of a sexual encounter occurring. However, the vignette information that was given to participants was ambiguous and was aimed to raise uncertainty about the fictional woman’s level of sexual interest and consent. Active tDCS may have served to heighten awareness of the mismatch between participants sexual goal and the potentially fraught scenario. In summary, men’s ratings of negative affect and, potentially, decision making may have been especially affected by vignette details that served to reduce the likelihood of their goal occurring while the DLPFC was stimulated.

However, the left DLPFC is only one neuroanatomical structure of many that were presently stimulated with the diffuse electric field induced by the bilateral electrode montage used here. Other cortical structures, such as the ventromedial prefrontal cortex, may have feasibly been impacted by stimulation and influenced individuals’ imagined affect state and decision-making in these hypothetical social scenarios. Instead of assigning complete responsibility of the current effects to the left DLPFC, we conclude here that bilateral stimulation of the PFC may have manipulated network-level activity related to reward processing. Indeed, prior investigations have demonstrated that neuromodulation effects can extend beyond their immediate stimulation site, with evidence that electrical stimulation of the PFC can impart effects on nearby cortical structures (e.g. the dorsal anterior cingulate cortex; Longe et al. 2009; Weber et al. 2014), as well as subcortical regions distal to the stimulation site (Meyer et al. 2019). Operating from this understanding, anodal tDCS may have caused the PFC – and the information encoded by its activity – to have a greater projective impact on relevant reward processing networks, thus producing these interaction effects. Further work that can disambiguate the role of stimulation on specific cortical structures (e.g. the left DLPFC) represent fruitful ground for future hypothesis development and investigation with adjacent neuroscientific methods (e.g. fMRI and EEG).

Elsewhere, there is evidence that tDCS delivered to the PFC can modify the motivational balance of approach/avoidance affect and behaviors, with the left-hemisphere specifically supporting forward, risky, and/or aggressive behavior across studies (Kelley et al. 2017, Ohmann et al. 2018). Interestingly, some of these tDCS investigations have highlighted the important contribution of situational context, as the effect of stimulation on negative emotion (e.g. jealousy and anger) and aggressive behavior have depended upon social circumstances like interpersonal provocation or social exclusion, respectively (see Hortensius et al. 2012 & Kelley et al. 2015). The present effects are well-aligned with these prior findings, and suggest that men’s negative affective reactions, and potentially, behavioral intentions, became more prominently accorded to women’s refusals or lack thereof when anodal tDCS was delivered to the left PFC.

Next, women’s responses to men’s advances in the vignettes – of which included explicit refusals or ambiguous behavior – were consistently found to influence each study outcome. Elsewhere, women’s hypothetical objections (or lack thereof) have been demonstrated to similarly influence men’s responses to vignette paradigms (Anduze et al. 2024, Lofgreen et al. 2021, McKinnon et al. 2025). A compelling interpretation of these converging findings is revealed through the application of sexual script theory (Wiederman 2005), which contends that sexual interactions are contextualized within social cognitions that individuals subscribe to. Young men, in particular, may accept heteronormative sexual scripts that serve to justify forward or aggressive sexual behavior. For example, men may interpret themselves and their behaviors as being primarily sexually-motivated (Sakaluk et al. 2014) and believe that they are responsible for initiating sexual overtures. Correspondingly, they may believe that women must correspondingly act as wary gatekeepers of sexual behavior, clearly refusing those who are unworthy of partnership. This commonplace script may be closely applicable to the current paradigm as this sample of heterosexual males may have oriented to the encounter as one wherein a man attempts to demonstrate his masculinity and sexual prowess to a woman in hopes that she would comply with his sexual advances.

Through the lens of sexual script theory, a refusal in the current work may have been indicative to this sample that they failed to pass muster for sexual behavior. It may have even been perceived as a personal rebuke of the individual and their masculinity, thereby producing frustration and negative affect (Bushman et al. 2003). Conversely, a woman’s ambiguous or passive behavior may have been interpreted as a lack of objection and/or silent compliance. This perspective may also clarify the relative unimportance of other factors in models, as men may have primarily attended to factors that were most immediately relevant to sexual behavior based on heteronormative scripts (e.g. women’s responses and the level of sexual behavior already occurring). Lastly, stimulation interacted with women’s responses and it is unclear what kind of simultaneous cognitive effects that this may have had on men. It may be that stimulation caused men to attend even more closely towards factors that they believed to be stereotypically associated with or salient to their sexual goals. Under such a scenario, men’s awareness of overt situational cues such as a woman’s refusal (or lack thereof) may have been enhanced by tDCS. In contrast, other contextual details that appeared relevant to the sample’s consent appraisals (i.e. the level of alcohol use or intimacy attained in the scenario) may have lacked sufficient cognitive salience to interact with tDCS to direct their negative affective reactions or decision making. Alternatively, stimulation may have simply changed the intensity of men’s behavioral and negative affective responding in provocative scenarios such as a sexual refusal. Future study that can investigate how PFC manipulations affect the processing of sexual information in the moment may clarify the cognitive-affective determinants of aggressive decision making.

Overall, these results highlight the potential of prevention efforts that aim to improve men’s cognitive processing of sexual scenarios. An interventional focus that can modify how men use situational cues (e.g. women’s communication) to inform their decision making has elsewhere been suggested to impact sexual decision making (Farris et al. 2008, Treat et al. 2016). Current findings demonstrate that modifying the activity of the PFC enhances the relevance of situational cues (e.g. women’s refusals) on men’s sexual decision making. Interventions that strengthen the salience of women’s communication to men during sexual negotiations may thus be intervention targets. A key component of such an approach may be to improve men’s affective and behavioral sensitivity to signals of disinterest. Additionally, men in the current work were more likely to proceed with sexual behavior in ambiguous scenarios lacking affirmative consent, signaling the importance of education that the absence of a refusal may be necessary but ultimately insufficient for determining consent. Individuals likely to respond to this sort of preventative training may include those with inadequately developed social skills or who are sexually inexperienced (Farris et al. 2008). In contrast, especially impulsive or aggressive individuals may not be ideal candidates, as they may proceed with sexual behavior regardless of women’s refusals or apparent consequences of transgression.

Further research is warranted to clarify the replicability and specificity of the observed effects. An immediate next step is to evaluate the effects of neuromodulation under different operations, including application of a contralateral electrode montage (e.g. anode placed over the right PFC and cathode placed on the left PFC) and by exploring more localized electrical fields. This latter point may be a critical direction, as the diffuse tDCS approach used here limits claims regarding the relation of particular cortical regions (e.g. the left DLPFC) or that of higher-order brain networks (e.g. the central executive) on sexual decision making. Alternatively, it appears worthwhile to use neuroimaging to examine the functional connectivity of the PFC and its association with men’s sexual decision making. Lastly, investigators could explore whether the present effects can rescue cognitive control that is impaired during the administration of alcohol or other substances, thereby clarifying the extent to which these processes are malleable during periods of inebriation. Regardless of the methodology used, delineating the neurocognitive processes underlying appraisals of sexual information and the ultimate selection of sexual behavior may guide intervention development.

A conditional and small effect of neuromodulation was found on hypothetical sexual behavior in this work. Although, this effect did not survive correction for multiple comparisons it appeared to further calibrate men’s decision making to women’s communication. Due to both the uncertainty regarding this finding and the experimental nature of this work, it appears pertinent to state that it is not indicated that tDCS be used as a preventative or adjunctive measure for altering sexual decision making or behavior at this time. Notwithstanding the impracticality of such an approach, brain stimulation may have led to an increase in anticipated negative affect, which is known to associate with sexually aggressive behavior (Bushman et al. 2003). It is unclear if a clinical application of tDCS could inadvertently contribute to aggressive behavior, thereby predisposing individuals to become frustrated or incensed during sexual encounters. It is presently unlikely, however, that the current use of tDCS conferred any lasting risk in participants’ cognitions or behavior in real-world scenarios, given the short-lived impact of a single session of tDCS on cortical activity (Monte-Silva et al. 2010). Furthermore, the effects of tDCS are state-dependent on endogenous synaptic activity (Esmaeilpour et al. 2018) and presently differed according to vignette characteristics. Therefore, it is uncertain what effect tDCS might have on sexual behavior when paired with therapeutic interventions. Prior to any clinical application of tDCS for sexual behavior, we encourage further experimentation that examines if and how sexual decision making is influenced by tDCS directed at the PFC and its potential interactions with situational context and dispositional traits.

The overall experimental design is next worthy of consideration. The use of tDCS is novel in this field and experimentally links PFC activity to sexual decision making. Compared with other experimental methodologies in this area of research (e.g. cognitive reappraisal training), tDCS may circumvent practical issues – such as whether participants have the ability or interest in modifying their sexual cognitions or behavior – via a direct PFC manipulation. However, there are also shortcomings to this study’s experimental procedures. First, as discussed earlier, the bifrontal montage used here induced a diffuse electrical field that may confound the interpretations regarding specific cortical structures (e.g. the DLPFC). More critically, this study aimed to deliver 2.0 mA of stimulation, but a small set of individuals in the active condition preferred to receive 1.5 mA and 1.0 mA intensities based on comfortability. The effects of differing doses of tDCS are known to be non-linear and it is unclear whether individuals who received a lower dose of stimulation presently differed in their affective and behavioral reactions to the vignettes. But the impact of this limitation on this study’s results appears minimal, as sensitivity analyses that excluded these individuals found that the overall pattern of effects holds, despite the smaller analytic sample.

A final limitation regards the delivery of tDCS in two phases, first consisting of an initial 10-minute offline phase followed by approximately 15–20 minutes of online stimulation while participants completed study vignettes. Although this approach was used to enhance the likelihood of active neuromodulation effects during task completion, it is unclear how vignette responding might differ if subjects received only offline or varying forms of online stimulation (e.g. with different simultaneous tasks). A promising direction for further research may be to assess the impact of online vs. offline stimulation alongside the practice of clinical techniques (e.g. cognitive reappraisal) to reveal the relevance of these parameters on participant outcomes.

Next, this study’s vignette paradigm experimentally controlled for how information regarding the date was presented and was thus advantageous for studying cognitive-affective processes. Although the vignette approach does not exactly mirror *in vivo* experiences, it enables measurement of participant cognitions and decision making without the overlapping confounds of real-world influences such as alcohol use, relational difficulties, and sexual arousal. However, it was not without its shortcomings as the vignette approach used here required a robust statistical approach to account for between- and within-subjects factors. Although the generalized linear mixed model is well suited to handle such a data frame, the specification of multiple fixed effects and their interaction terms enhanced the potential for multicollinearity and Type 1 error. Additionally, this study presented situational elements in vignettes in a fixed order, where a night out gradually proceeds before an ambiguous sexual encounter begins. Other research has demonstrated that the order in which situational elements are presented affects appraisals of sexual consent (Willis and Jozkowski 2022) and it is unclear how the preset order of situational information in these vignettes may have influenced study outcomes. Future investigators are encouraged to consider simpler experimental approaches that are capable of testing cognitive mediators of the neuromodulation approaches as well as to test whether the effects presently observed replicate or generalize to other dating violence paradigms.

A final series of considerations pertains to the sample of participants collected here. This study recruited a sample of young men who were primarily university students and identifying as White. Furthermore, this work sought to evaluate the decision making of men in the context of a ‘hookup’ scenario after a night out that more closely aligns with young adult dating patterns. The findings observed here are thus limited in their generalizability to other populations (e.g. individuals with a history of sexual offending) and other contexts relevant to sexual aggression (e.g. intimate partner violence). Additionally, this sample overall reported limited lifetime sexual experiences, which may be consistent with recent trends of relatively lower levels of sexual activity among the youngest men in our society (Ueda et al. 2020). Practically, it will be important for future research with young men to evaluate how their lifetime sexual experiences shape their appraisals and reactions to sexual scenarios. It is recommended that subsequent investigations closely tailor sexual outcome measures to the types of interpersonal and sexual encounters that young men are regularly experiencing.

## Conclusion

The study of dating violence has lacked empirical research that tests how the PFC influences sexual decision making. Presently, tDCS was delivered to the PFC as an experimental manipulation to bridge this gap in the literature. Women’s communication interacted with tDCS to affect the hypothetical likelihood of negative affective responses. Several future directions for research, including expanding the use of neuroscience in the field of dating violence appear warranted as well as the exploration of cognitive interventions that could support positive sexual functioning for men.

## Supplementary Material

Supplementary_materials_kvag003

## Data Availability

Data are available via the following link: https://osf.io/j2nre/.

## Footnotes

1 Time between study visits was not found to be significantly associated with study outcomes (*p*’s > 0.05) based on the results of additional GLM models that included time as a covariate (See Supplementary Table 10).

2 Participants that experienced discomfort above 4/10 on a pain visual analogue scale during a stimulation tolerability test were afforded the opportunity to complete stimulation at lower intensities (i.e. 1.5 mA and 1.0 mA). Individuals who did not tolerate 1.0 mA based on these criteria were not asked to complete any further stimulation procedures. GLM models were also conducted without individuals that did not tolerate 2.0 mA stimulation in the active condition, the pattern of findings was not found to differ (See Supplementary Tables 11, 12, 13, and 14).

3 See Supplementary Table 15 for reporting of stimulation side effects.
