# Supplementary material for: An examination of the influence of prefrontal cortical brain stimulation on sexual decision making
Source: Oxf Open Neurosci. 2026 May 15;5:kvag003. doi: 10.1093/oons/kvag003 (PMC13225266; doi:10.1093/oons/kvag003)
Supplement: Supplementary_materials_kvag003 [file supplementary_materials_kvag003.zip › SupplementaryMaterial_R1.docx]

**Supplementary Table 1**

*Vignette Response Measures*

| Category/Item | Scale |
| --- | --- |
| Affect State Outcomes | |
| Positive Affect: Excited, Happy, Strong, Manly, Determined, Sexually Aroused | 1 (*Not at all*) to 7 (*Extremely)* |
| Negative Affect: Angry, Disappointed, Frustrated, Irritated, Resentful, Disgusted | 1 (*Not at all*) to 7 (*Extremely)* |
| Likelihood of Sexually Advancing Outcome | |
| Continue engaging in the initial activity, but stop trying to advance it to a more intimate level | 1 (*Very unlikely*) to 7 (*Very likely)* |
| Share your romantic feelings towards your date and try to advance the activity to a more intimate level | 1 (*Very unlikely*) to 7 (*Very likely)* |
| Continue engaging in the initial activity. And try to verbally convince your date to advance the activity to a more intimate level | 1 (*Very unlikely*) to 7 (*Very likely)* |
| Continue engaging in the initial activity. And try again in a few minutes to advance it to a more intimate level | 1 (*Very unlikely*) to 7 (*Very likely)* |
| Remind your date that they want to have sex based on their behavior tonight | 1 (*Very unlikely*) to 7 (*Very likely)* |
| Continue advancing the level of intimacy – tonight's your lucky night | 1 (*Very unlikely*) to 7 (*Very likely)* |
| Continue advancing the level of intimacy by ignoring your date | 1 (*Very unlikely*) to 7 (*Very likely)* |
| Continue advancing the level of intimacy by using some degree of physical force | 1 (*Very unlikely*) to 7 (*Very likely)* |

*Note*. For affective responses, participants were prompted: “Again, imagine yourself in this situation. To what extent does this situation make you feel:”. For behavioral likelihood responses, participants were prompted: “Given the information, how likely is it that you would respond to the situation in the following ways?”

**Supplementary Table 2**

*Consent GLMM with Situational Factors*

| Model Term | Coefficient | *SE* | *t* | *p* | 95% CI |
| --- | --- | --- | --- | --- | --- |
| Intercept | 0.71 | 0.16 | 4.55 | <0.001 | [0.4, 1.01] |
| Consent at Baseline | 0.17 | 0.02 | 8.86 | <0.001 | [0.13, 0.2] |
| Attempted Emotional Vulnerability (No vs. Yes) | 0.10 | 0.08 | 1.39 | 0.167 | [-0.04, 0.25] |
| Meeting Source (Dating App) | 0.00 | 0.11 | 0.01 | 0.992 | [-0.21, 0.21] |
| Meeting Source (Bar) | -0.03 | 0.10 | -0.28 | 0.780 | [-0.23, 0.17] |
| Meeting Source (Through Friend) | -0.08 | 0.10 | -0.80 | 0.425 | [-0.27, 0.11] |
| Feel Close to Date (No vs. Yes) | 0.01 | 0.07 | 0.09 | 0.927 | [-0.14, 0.15] |
| Sex History (No History of Prior  Sex Specified) | 0.03 | 0.11 | 0.26 | 0.798 | [-0.18, 0.23] |
| Sex History (History of Prior Sex   Specified) | 0.05 | 0.10 | 0.54 | 0.588 | [-0.14, 0.25] |
| Sex History (Prior lower levels of   Sexual Behavior) | 0.10 | 0.10 | 0.91 | 0.363 | [-0.11, 0.3] |
| Vignette Specified Reader Used   Alcohol | -0.25 | 0.11 | -2.40 | 0.017 | [-0.46, -0.05] |
| Vignette Specified Date Used   Alcohol | -0.19 | 0.11 | -1.81 | 0.071 | [-0.4, 0.02] |
| Vignette Specified that Both Used   Alcohol | 0.25 | 0.15 | 1.71 | 0.088 | [-0.04, 0.54] |
| Sexual Intimacy (None, Attempt at   Kissing) | -0.47 | 0.10 | -4.58 | <0.001 | [-0.67, -0.27] |
| Sexual Intimacy (Kissing, Attempt   at Undressing) | -0.21 | 0.11 | -1.93 | 0.054 | [-0.42, 0] |
| Sexual Intimacy (Oral Sex,   Attempt at Intercourse) | -0.17 | 0.10 | -1.64 | 0.102 | [-0.38, 0.03] |
| Refusal Response (Compared with   Passive/Ambiguous Response) | -0.21 | 0.07 | -2.86 | 0.005 | [-0.36, -0.07] |

**Supplementary Table 3**

*Consent GLMM with tDCS Condition*

| Model Term | Coefficient | *SE* | *t* | *p* | 95% CI |
| --- | --- | --- | --- | --- | --- |
| Intercept | 0.86 | 0.13 | 6.57 | <0.001 | [0.6, 1.12] |
| Sham tDCS Condition (Compared   with Active) | -0.06 | 0.18 | -0.35 | 0.730 | [-0.42, 0.29] |
| Consent at Baseline | 0.17 | 0.02 | 9.03 | <0.001 | [0.13, 0.2] |
| Sexual Intimacy (None, Attempt at   Kissing) | -0.42 | 0.14 | -3.09 | 0.002 | [-0.7, -0.15] |
| Sexual Intimacy (Kissing, Attempt   at Undressing) | -0.28 | 0.15 | -1.92 | 0.057 | [-0.58, 0.01] |
| Sexual Intimacy (Oral Sex,   Attempt at Intercourse) | -0.32 | 0.16 | -2.06 | 0.041 | [-0.63, -0.01] |
| Refusal Response (Compared with   Passive/Ambiguous Response) | -0.19 | 0.11 | -1.77 | 0.077 | [-0.39, 0.02] |
| Vignette Specified Reader Used   Alcohol | -0.16 | 0.11 | -1.55 | 0.123 | [-0.37, 0.04] |
| Refusal Response (Compared with   Passive/Ambiguous   Response)*tDCS Condition | -0.08 | 0.15 | -0.52 | 0.603 | [-0.37, 0.21] |
| Sexual Intimacy (None, Attempt at   Kissing)*tDCS Condition | -0.14 | 0.21 | -0.69 | 0.490 | [-0.55, 0.26] |
| Sexual Intimacy (Kissing, Attempt   at Undressing)*tDCS   Condition | 0.13 | 0.21 | 0.63 | 0.532 | [-0.29, 0.55] |
| Sexual Intimacy (Oral Sex,   Attempt at Intercourse)*tDCS   Condition | 0.21 | 0.21 | 1.01 | 0.312 | [-0.2, 0.62] |
| Vignette Specified Reader Used   Alcohol*tDCS Condition | 0.06 | 0.15 | 0.43 | 0.667 | [-0.23, 0.35] |

**Supplementary Table 4**

*Negative Affect GLMM with Situational Factors*

| Model Term | Coefficient | *SE* | *t* | *p* | 95% CI |
| --- | --- | --- | --- | --- | --- |
| Intercept | 1.67 | 0.08 | 21.69 | <0.001 | [1.52, 1.82] |
| Negative Affect at Baseline | 0.02 | 0.003 | 7.20 | <0.001 | [0.02, 0.03] |
| Attempted Emotional   Vulnerability (No vs. Yes) | 0.03 | 0.04 | 0.92 | 0.357 | [-0.04, 0.1] |
| Meeting Source (Dating App) | 0.07 | 0.05 | 1.43 | 0.154 | [-0.03, 0.17] |
| Meeting Source (Bar) | 0.02 | 0.05 | 0.44 | 0.657 | [-0.07, 0.12] |
| Meeting Source (Through Friend) | 0.01 | 0.05 | 0.27 | 0.789 | [-0.08, 0.1] |
| Feel Close to Date (No vs. Yes) | 0.004 | 0.04 | 0.11 | 0.912 | [-0.07, 0.07] |
| Sex History (No History of Prior   Sex Specified) | 0.01 | 0.05 | 0.30 | 0.766 | [-0.08, 0.11] |
| Sex History (History of Prior Sex   Specified) | 0.04 | 0.05 | 0.84 | 0.400 | [-0.05, 0.13] |
| Sex History (Prior lower levels of   Sexual Behavior) | -0.02 | 0.05 | -0.43 | 0.665 | [-0.12, 0.08] |
| Vignette Specified Reader Used   Alcohol | -0.03 | 0.05 | -0.52 | 0.606 | [-0.12, 0.07] |
| Vignette Specified Date Used   Alcohol | 0.01 | 0.05 | 0.23 | 0.815 | [-0.09, 0.11] |
| Vignette Specified that Both Used   Alcohol | 0.09 | 0.07 | 1.36 | 0.175 | [-0.04, 0.23] |
| Sexual Intimacy (None, Attempt at   Kissing) | 0.09 | 0.05 | 1.80 | 0.074 | [-0.01, 0.18] |
| Sexual Intimacy (Kissing, Attempt   at Undressing) | -0.02 | 0.05 | -0.41 | 0.682 | [-0.12, 0.08] |
| Sexual Intimacy (Oral Sex,   Attempt at Intercourse) | -0.01 | 0.05 | -0.26 | 0.792 | [-0.11, 0.08] |
| Refusal Response (Compared with   Passive/Ambiguous Response) | 0.29 | 0.03 | 8.53 | <0.001 | [0.23, 0.36] |

**Supplementary Table 5**

*Negative Affect GLMM with tDCS Condition*

| Model Term | Coefficient | *SE* | *t* | *p* | 95% CI |
| --- | --- | --- | --- | --- | --- |
| Intercept | 1.78 | 0.05 | 35.36 | <0.001 | [1.68, 1.88] |
| Sham tDCS Condition (Compared   with Active) | 0.06 | 0.05 | 1.08 | 0.282 | [-0.05, 0.17] |
| Negative Affect at Baseline | 0.02 | 0.003 | 7.08 | <0.001 | [0.02, 0.03] |
| Refusal Response (Compared with   Passive/Ambiguous Response) | 0.39 | 0.05 | 8.56 | <0.001 | [0.3, 0.48] |
| Woman's Communication*tDCS   Condition | -0.19 | 0.06 | -3.09 | 0.002 | [-0.32, -0.07] |

**Supplementary Table 6**

*Positive Affect GLMM with Situational Factors*

| Model Term | Coefficient | *SE* | *t* | *p* | 95% CI |
| --- | --- | --- | --- | --- | --- |
| Intercept | 2.70 | 0.11 | 24.22 | <0.001 | [2.48, 2.92] |
| Positive Affect at Baseline | 0.02 | 0.003 | 8.50 | <0.001 | [0.02, 0.03] |
| Attempted Emotional Vulnerability   (No vs. Yes) | 0.07 | 0.05 | 1.52 | 0.130 | [-0.02, 0.17] |
| Meeting Source (Dating App) | -0.09 | 0.07 | -1.36 | 0.174 | [-0.23, 0.04] |
| Meeting Source (Bar) | -0.03 | 0.07 | -0.38 | 0.704 | [-0.16, 0.11] |
| Meeting Source (Through Friend) | -0.09 | 0.06 | -1.37 | 0.172 | [-0.21, 0.04] |
| Feel Close to Date (No vs. Yes) | -0.11 | 0.05 | -2.32 | 0.021 | [-0.21, -0.02] |
| Sex History (No History of Prior   Sex Specified) | -0.06 | 0.07 | -0.81 | 0.419 | [-0.19, 0.08] |
| Sex History (History of Prior Sex   Specified) | -0.08 | 0.06 | -1.28 | 0.203 | [-0.21, 0.04] |
| Sex History (Prior lower levels of   Sexual Behavior) | 0.04 | 0.07 | 0.59 | 0.554 | [-0.09, 0.17] |
| Vignette Specified Reader Used   Alcohol | -0.06 | 0.07 | -0.86 | 0.388 | [-0.19, 0.08] |
| Vignette Specified Date Used   Alcohol | -0.03 | 0.07 | -0.47 | 0.639 | [-0.17, 0.1] |
| Vignette Specified that Both Used   Alcohol | -0.04 | 0.09 | -0.46 | 0.645 | [-0.23, 0.14] |
| Sexual Intimacy (None, Attempt at   Kissing) | -0.29 | 0.07 | -4.30 | <0.001 | [-0.42, -0.15] |
| Sexual Intimacy (Kissing, Attempt   at Undressing) | -0.23 | 0.07 | -3.23 | 0.001 | [-0.38, -0.09] |
| Sexual Intimacy (Oral Sex,   Attempt at Intercourse) | -0.09 | 0.07 | -1.37 | 0.171 | [-0.23, 0.04] |
| Refusal Response (Compared with   Passive/Ambiguous Response) | -0.27 | 0.05 | -5.69 | <0.001 | [-0.36, -0.17] |

**Supplementary Table 7**

*Positive Affect GLMM with tDCS Condition*

| Model Term | Coefficient | *SE* | *t* | *p* | 95% CI |
| --- | --- | --- | --- | --- | --- |
| Intercept | 2.57 | 0.10 | 25.20 | <0.001 | [2.37, 2.77] |
| Sham tDCS Condition (Compared   with Active) | 0.01 | 0.13 | 0.12 | 0.905 | [-0.23, 0.26] |
| Positive Affect at Baseline | 0.03 | 0.003 | 8.83 | <0.001 | [0.02, 0.03] |
| Feel Close to Date (No) | -0.15 | 0.07 | -2.18 | 0.030 | [-0.28, -0.01] |
| Sexual Intimacy (None, Attempt at   Kissing) | -0.17 | 0.09 | -1.89 | 0.060 | [-0.34, 0.01] |
| Sexual Intimacy (Kissing, Attempt at   Undressing) | -0.09 | 0.10 | -0.93 | 0.355 | [-0.29, 0.1] |
| Sexual Intimacy (Oral Sex, Attempt   at Intercourse) | 0.03 | 0.10 | 0.26 | 0.795 | [-0.17, 0.23] |
| Refusal Response (Compared with   Passive/Ambiguous Response) | -0.34 | 0.06 | -5.27 | <0.001 | [-0.47, -0.21] |
| Feel Close to Date (No)*Sham tDCS   Condition (Compared with   Active) | 0.06 | 0.10 | 0.61 | 0.541 | [-0.13, 0.25] |
| Sexual Intimacy (None, Attempt at   Kissing)*tDCS Condition | -0.23 | 0.13 | -1.69 | 0.093 | [-0.49, 0.04] |
| Sexual Intimacy (Kissing, Attempt at   Undressing)*tDCS Condition | -0.25 | 0.14 | -1.75 | 0.081 | [-0.52, 0.03] |
| Sexual Intimacy (Oral Sex, Attempt   at Intercourse)*tDCS Condition | -0.22 | 0.14 | -1.62 | 0.107 | [-0.49, 0.05] |
| Woman's Communication*tDCS   Condition | 0.13 | 0.09 | 1.47 | 0.142 | [-0.05, 0.31] |

**Supplementary Table 8**

*Decision Making GLMM with Situational Factors*

| Model Term | Coefficient | *SE* | *t* | *p* | 95% CI |
| --- | --- | --- | --- | --- | --- |
| Intercept | 2.37 | 0.09 | 27.19 | <0.001 | [2.2, 2.54] |
| Decision Making at Baseline | 0.03 | 0.003 | 8.62 | <0.001 | [0.02, 0.04] |
| Attempted Emotional Vulnerability   (No vs. Yes) | 0.02 | 0.04 | 0.51 | 0.610 | [-0.05, 0.09] |
| Meeting Source (Dating App) | 0.00 | 0.05 | 0.02 | 0.983 | [-0.1, 0.1] |
| Meeting Source (Bar) | 0.01 | 0.05 | 0.21 | 0.836 | [-0.09, 0.11] |
| Meeting Source (Through Friend) | -0.02 | 0.05 | -0.50 | 0.621 | [-0.11, 0.07] |
| Feel Close to Date (No vs. Yes) | 0.00 | 0.04 | 0.13 | 0.898 | [-0.07, 0.08] |
| Sex History (No History of Prior   Sex Specified) | -0.07 | 0.05 | -1.34 | 0.182 | [-0.17, 0.03] |
| Sex History (History of Prior Sex   Specified) | -0.08 | 0.05 | -1.57 | 0.117 | [-0.17, 0.02] |
| Sex History (Prior lower levels of   Sexual Behavior) | -0.03 | 0.05 | -0.59 | 0.559 | [-0.13, 0.07] |
| Vignette Specified Reader Used   Alcohol | -0.08 | 0.05 | -1.52 | 0.129 | [-0.18, 0.02] |
| Vignette Specified Date Used   Alcohol | -0.05 | 0.05 | -0.96 | 0.340 | [-0.15, 0.05] |
| Vignette Specified that Both Used   Alcohol | 0.04 | 0.07 | 0.54 | 0.588 | [-0.1, 0.17] |
| Sexual Intimacy (None, Attempt at   Kissing) | -0.12 | 0.05 | -2.52 | 0.012 | [-0.22, -0.03] |
| Sexual Intimacy (Kissing, Attempt   at Undressing) | -0.07 | 0.05 | -1.27 | 0.205 | [-0.17, 0.04] |
| Sexual Intimacy (Oral Sex,   Attempt at Intercourse) | -0.01 | 0.05 | -0.24 | 0.808 | [-0.11, 0.09] |
| Refusal Response (Compared with   Passive/Ambiguous Response) | -0.13 | 0.03 | -3.74 | <0.001 | [-0.2, -0.06] |

|  |  |  |  |
| --- | --- | --- | --- |

**Supplementary Table 9**

*Decision Making GLMM with tDCS Condition*

| Model Term | Coefficient | *SE* | *t* | *p* | 95% CI |
| --- | --- | --- | --- | --- | --- |
| Intercept | 2.31 | 0.07 | 31.72 | <0.001 | [2.16, 2.45] |
| Sham tDCS Condition (Compared   with Active) | -0.03 | 0.09 | -0.35 | 0.729 | [-0.2, 0.14] |
| Decision Making at Baseline | 0.03 | 0.003 | 8.80 | <0.001 | [0.02, 0.03] |
| Sexual Intimacy (None, Attempt at   Kissing) | -0.10 | 0.06 | -1.65 | 0.099 | [-0.23, 0.02] |
| Sexual Intimacy (Kissing, Attempt at   Undressing) | -0.02 | 0.07 | -0.30 | 0.766 | [-0.16, 0.12] |
| Sexual Intimacy (Oral Sex, Attempt   at Intercourse) | 0.02 | 0.07 | 0.30 | 0.766 | [-0.12, 0.17] |
| Refusal Response (Compared with   Passive/Ambiguous Response) | -0.20 | 0.05 | -4.37 | <0.001 | [-0.3, -0.11] |
| Sexual Intimacy (None, Attempt at   Kissing)*tDCS Condition | -0.02 | 0.10 | -0.22 | 0.828 | [-0.22, 0.17] |
| Sexual Intimacy (Kissing, Attempt at   Undressing)*tDCS Condition | -0.07 | 0.10 | -0.73 | 0.465 | [-0.27, 0.13] |
| Sexual Intimacy (Oral Sex, Attempt   at Intercourse)*tDCS Condition | -0.06 | 0.10 | -0.58 | 0.561 | [-0.25, 0.14] |
| Woman's Communication*tDCS   Condition | 0.15 | 0.07 | 2.25 | 0.025 | [0.02, 0.28] |

**Supplementary Table 10**

| GLM Model | *F* | *p* |
| --- | --- | --- |
| Consent | 0.20 | 0.658 |
| Positive Affect | 2.08 | 0.150 |
| Negative Affect | 0.01 | 0.922 |
| Decision Making | 0.27 | 0.870 |

*Testing Days Between Study Visits as a Covariate*

*Note.* These models used the full suite of predictors from the tDCS GLM models and additionally included the days between study visits as a covariate to determine if it significantly affected the study outcome or modified the relations between the tDCS predictors and study outcomes. The covariate was not found to be significant related to the outcome in any model and did not modify the association of the tDCS predictors or interaction terms with outcomes.

**Supplementary Table 11**

*Sensitivity Analysis of Consent GLMM with tDCS Condition*

| Model Term | Coefficient | *SE* | *t* | *p* | 95% CI |
| --- | --- | --- | --- | --- | --- |
| Intercept | 0.88 | 0.15 | 6.04 | <0.001 | [0.6, 1.17] |
| Sham tDCS Condition (Compared   with Active) | -0.06 | 0.19 | -0.33 | 0.745 | [-0.44, 0.32] |
| Consent at Baseline | 0.15 | 0.02 | 7.84 | <0.001 | [0.12, 0.19] |
| Sexual Intimacy (None, Attempt at   Kissing) | -0.54 | 0.16 | -3.44 | 0.001 | [-0.85, -0.23] |
| Sexual Intimacy (Kissing, Attempt   at Undressing) | -0.27 | 0.17 | -1.55 | 0.122 | [-0.62, 0.07] |
| Sexual Intimacy (Oral Sex,   Attempt at Intercourse) | -0.32 | 0.17 | -1.83 | 0.069 | [-0.66, 0.03] |
| Refusal Response (Compared with   Passive/Ambiguous Response) | -0.27 | 0.12 | -2.30 | 0.022 | [-0.50, -0.04] |
| Vignette Specified Reader Used   Alcohol | -0.10 | 0.12 | -0.81 | 0.418 | [-0.34, 0.14] |
| Refusal Response (Compared with   Passive/Ambiguous   Response)*tDCS Condition | 0.01 | 0.15 | 0.05 | 0.964 | [-0.30, 0.31] |
| Sexual Intimacy (None, Attempt at   Kissing)*tDCS Condition | -0.01 | 0.22 | -0.06 | 0.952 | [-0.45, 0.42] |
| Sexual Intimacy (Kissing, Attempt   at Undressing)*tDCS   Condition | 0.12 | 0.23 | 0.51 | 0.614 | [-0.34, 0.57] |
| Sexual Intimacy (Oral Sex,   Attempt at Intercourse)*tDCS   Condition | 0.22 | 0.22 | 0.99 | 0.326 | [-0.22, 0.66] |
| Vignette Specified Reader Used   Alcohol*tDCS Condition | -0.01 | 0.16 | -0.09 | 0.931 | [-0.33, 0.30] |

*Note.* This sensitivity analysis excluded individuals in the active condition who did not receive 2.0mA stimulation.

**Supplementary Table 12**

*Sensitivity Analysis of Negative Affect GLMM with tDCS Condition*

| Model Term | Coefficient | *SE* | *t* | *p* | 95% CI |
| --- | --- | --- | --- | --- | --- |
| Intercept | 1.75 | 0.06 | 31.11 | <0.001 | [1.64, 1.87] |
| Sham tDCS Condition (Compared   with Active) | 0.07 | 0.06 | 1.17 | 0.245 | [-0.05, 0.18] |
| Negative Affect at Baseline | 0.02 | 0.004 | 6.97 | <0.001 | [0.02, 0.03] |
| Refusal Response (Compared with   Passive/Ambiguous Response) | 0.39 | 0.05 | 7.35 | <0.001 | [0.29, 0.50] |
| Woman's Communication*tDCS   Condition | -0.20 | 0.07 | -2.94 | 0.004 | [-0.34, -0.07] |

*Note.* This sensitivity analysis excluded individuals in the active condition who did not receive 2.0mA stimulation.

**Supplementary Table 13**

*Sensitivity Analysis of Positive Affect GLMM with tDCS Condition*

| Model Term | Coefficient | *SE* | *t* | *p* | 95% CI |
| --- | --- | --- | --- | --- | --- |
| Intercept | 2.57 | 0.11 | 22.91 | <0.001 | [2.35, 2.79] |
| Sham tDCS Condition (Compared   with Active) | 0.01 | 0.13 | 0.04 | 0.971 | [-0.25, 0.26] |
| Positive Affect at Baseline | 0.03 | 0.03 | 8.16 | <0.001 | [0.02, 0.03] |
| Feel Close to Date (No) | -0.05 | 0.08 | -0.60 | 0.548 | [-0.20, 0.10] |
| Sexual Intimacy (None, Attempt at   Kissing) | -0.35 | 0.10 | -3.47 | 0.001 | [-0.54, -0.15] |
| Sexual Intimacy (Kissing, Attempt at   Undressing) | -0.07 | 0.11 | -0.64 | 0.523 | [-0.29, 0.15] |
| Sexual Intimacy (Oral Sex, Attempt   at Intercourse) | -0.04 | 0.11 | -0.33 | 0.744 | [-0.26, 0.18] |
| Refusal Response (Compared with   Passive/Ambiguous Response) | -0.40 | 0.07 | -5.51 | <0.001 | [-0.54, -0.26] |
| Feel Close to Date (No)*Sham tDCS   Condition (Compared with   Active) | -0.05 | 0.10 | -0.46 | 0.647 | [-0.25, 0.15] |
| Sexual Intimacy (None, Attempt at   Kissing)*tDCS Condition | -0.05 | 0.14 | -0.32 | 0.749 | [-0.32, 0.23] |
| Sexual Intimacy (Kissing, Attempt at   Undressing)*tDCS Condition | -0.27 | 0.15 | -1.81 | 0.072 | [-0.56, 0.02] |
| Sexual Intimacy (Oral Sex, Attempt   at Intercourse)*tDCS Condition | -0.16 | 0.14 | -1.09 | 0.277 | [-0.44, 0.13] |
| Woman's Communication*tDCS   Condition | 0.20 | 0.09 | 2.07 | 0.040 | [0.01, 0.38] |

*Note.* This sensitivity analysis excluded individuals in the active condition who did not receive 2.0mA stimulation.

**Supplementary Table 14**

*Sensitivity Analysis of Decision Making GLMM with tDCS Condition*

| Model Term | Coefficient | *SE* | *t* | *p* | 95% CI |
| --- | --- | --- | --- | --- | --- |
| Intercept | 2.36 | 0.08 | 28.53 | <0.001 | [2.20, 2.52] |
| Sham tDCS Condition (Compared   with Active) | -0.02 | 0.09 | -0.26 | 0.796 | [-0.21, 0.16] |
| Decision Making at Baseline | 0.03 | 0.003 | 6.63 | <0.001 | [0.02, 0.03] |
| Sexual Intimacy (None, Attempt at   Kissing) | -0.14 | 0.07 | -1.93 | 0.055 | [-0.28, 0.003] |
| Sexual Intimacy (Kissing, Attempt at   Undressing) | 0.02 | 0.08 | 0.22 | 0.829 | [-0.14, 0.18] |
| Sexual Intimacy (Oral Sex, Attempt   at Intercourse) | 0.03 | 0.08 | 0.37 | 0.715 | [-0.13, 0.19] |
| Refusal Response (Compared with   Passive/Ambiguous Response) | -0.22 | 0.05 | -4.10 | <0.001 | [-0.32, -0.11] |
| Sexual Intimacy (None, Attempt at   Kissing)*tDCS Condition | -0.01 | 0.10 | -0.09 | 0.925 | [-0.20, 0.22] |
| Sexual Intimacy (Kissing, Attempt at  Undressing)*tDCS Condition | -0.12 | 0.11 | -1.05 | 0.294 | [-0.33, 0.10] |
| Sexual Intimacy (Oral Sex, Attempt   at Intercourse)*tDCS Condition | -0.07 | 0.11 | -0.67 | 0.506 | [-0.28, 0.14] |
| Woman's Communication*tDCS   Condition | 0.15 | 0.07 | 2.14 | 0.033 | [0.01, 0.29] |

*Note.* This sensitivity analysis excluded individuals in the active condition who did not receive 2.0mA stimulation.

**Supplementary Table 15**

*Side Effects of Stimulation*

| Side Effect | Sham Condition | | | Active Condition | | |
| --- | --- | --- | --- | --- | --- | --- |
|  | Frequency | Mean Intensity | Mean Duration (SD) | Frequency | Mean Intensity | Mean Duration (SD) |
| Tingling | 45 | 4.2 (1.94) | 5.13 (6.68) | 48 | 3.92 (1.51) | 18.5 (10.33) |
| Itching | 19 | 3.74 (1.48) | 6.79 (8.84) | 23 | 3.65 (1.64) | 13.87 (11.56) |
| Headache | 6 | 2.17 (1.47) | 4.08 (2.38) | 5 | 5 (1.58) | 12.2 (4.09) |
| Nausea | 2 | 6 (4.24) | 12 (11.31) | 0 | - | - |
| Burning Sensations | 18 | 4.61 (2.12) | 3.71 (7.48) | 33 | 4.21 (1.92) | 12 (11.24) |
| Sleepiness/Fatigue | 15 | 5.2 (1.9) | 10.27 (8.27) | 10 | 4.8 (2.04) | 14 (7.38) |
| Pain | 7 | 4 (1.63) | 8.71 (9.79) | 7 | 4.43 (1.72) | 17.86 (12.25) |

*Note.* Participants self-reported any sensations or side effects of stimulation that they experienced during the tDCS session. Participants rated how intense the experience using a scale of 1-10, with ten being extreme. Participants rated the duration of side effects in minutes. Stimulation side effects were routinely reviewed over the course of this study.

**Supplementary Information**

**Vignette Scenario Example:**

| “Through a mutual friend you met Emma, a girl who you think is very attractive. You have allowed yourself to be emotionally vulnerable and you feel very close to her. You've hung out with her previously but have not had sex before and you are hoping you will have the chance tonight. Over the night out, you had several alcoholic drinks, but she decided not to drink before you both returned to your home. After chatting for a while, you and Emma start kissing. After a few minutes of making out you're feeling really turned on, so you start to reach under Emma's shirt. In response, she pushes away from you.” |
| --- |

*Note*. A sample vignette is given here that depicts a stereotypical and fictional dating scenario. Vignette elements were constructed to reflect common rape myths related to perceptions of sexual consent, including how the pair met, their level of closeness and vulnerability, their prior sexual history, their level of alcohol use over an evening, their level of sexually intimate behavior, and the woman’s communication regarding sexual advances. Full vignette materials are available at MASKED.

**Eligibility Criteria:** To be eligible for the current project, it was required that participants be fluent in the English language. Other exclusion criteria included: Any individuals with a history of epilepsy or seizure disorder, uncontrolled migraines in the previous six months, a history of head trauma or medical implants in the head or neck, skin disorders or sensitive skin near the electrode sites (e.g., the forehead and hairline), treatment for a communicable skin infection over the previous twelve months, a history of clinically significant abnormalities on an electrocardiogram, or individuals with a pacemaker were ineligible to participate in the current study.

**Additional Demographic Details:** The sample varied in their sexual histories and relationship statuses. Sixty-one percent of the sample (*n* = 62) reported prior sexual intercourse with a woman and all identified as heterosexual males at screening with no history of sexual interaction with other men. One participant changed their reported sexual orientation from heterosexual at screening to queer at follow-up, but sensitivity analyses demonstrated that their inclusion in analyses did not alter the pattern of findings. Notably, the individual reported no history of sexual intercourse with a man. Individuals who reported previous sexual intercourse with women reported a median number of 1.50 lifetime sexual partners (range = 1-12). Additionally, more than half of the sample reported their relationship status as single (61.80%), with some individuals in self-described “serious” relationships (29.40%) and a smaller group in “casual” relationships (8.80%).
