# Supplementary material for: An examination of the influence of prefrontal cortical brain stimulation on sexual decision making
Source: Oxf Open Neurosci. 2026 May 15;5:kvag003. doi: 10.1093/oons/kvag003 (PMC13225266; doi:10.1093/oons/kvag003)
Supplement: Supplementary_materials_kvag003 [file supplementary_materials_kvag003.zip › Review history for OXFNSC-2026-006.R2.odt]

Review history for OXFNSC-2026-006.R2

Round 1 reviews
Reviewer 1
Recommendation – Minor Revision
Comments to the Author
Please see feedback on this manuscript below...
Abstract
- The abstract contains most of the necessary details, please add a few words onto, or change the wording at the end of the sentence that starts “Active stimulation significantly….” so that the novice reader can understand the direction of effect e.g., reduced negative affect after stimulation/neuromodulation

Page 5 line 38: The author mentions “downstream social behaviour” for the first time. For clarity please explain this phrase/term either earlier or briefly in/near that sentence. This is done in part in the next sentence but need further clarity and/or an example.

Participants and procedure

- Typo: “Procedures” should be “Procedure”

Page 6 line 6: Explain what you mean by “community members” as this can be interpreted to mean or specific closed community, a wider community or the general public. Perhaps a different terms such as “Members of the public” would be more suitable here.

Page 6 from line 15: A good level of eligibility criteria are mentioned. These are typical/conventional of neuromodulation experiments - the novice reader might not know this. It could improve your writing if you say that these are the convention for neuromodulation.


Page 7 line 15: Explain what you mean by “did not tolerate the stimulation procedures” it is likely that you mean minor itchiness or slight skin irritation/sensitivity.

Page 8 line 11: The authors mention that some participants received 2mA, some 1.5mA and some 1mA - please explain why this is the case. This is a big limitation for the interpretation of results. e.g., a 2mA intensity could produce very different results to 1mA.

Page 8 line 13: The idea about asking participants which type of stimulation/sham they received was a nice idea. The authors needs to explain whether the participants were novices or not to neuromodulation - if they were then they would not understand the relevant terms.

tdCS procedure
- the authors clearly mention 2mA intensity. Above in the previous section 1.5mA and 1mA are also mentioned. If 2mA was used then the earlier section needs correcting. If 2mA was used (as mentioned in the tDCS procedure) then the other intensities need to be mentioned here too with a clear justification for why there were 3 intensity levels.

- it sounds as if ‘offline stimulation’ was used. Please justify why this was used compared to ‘online stimulation’ (i.e., task during stimulation). There is plenty of literature that can be used for this.

- No ramp-up/down period is mentioned. If this was used please mention it, if not say it was not used.

- Add the duration of the task. This is important due to the duration of the effect/influence of offline stimulation.

Discussion section
The discussion section is generally well written although the following could be considered to improve…

- The issue mentioned above around the different current intensities needs to be addressed as a limitation of this work.  


Reviewer 2
Recommendation – Minor Revision
Comments to the Author:
Manuscript ID: OXFNSC-2026-006
Title: An Examination of the Influence of Prefrontal Cortical Brain Stimulation on Sexual Decision Making
This study investigated the neurocognitive mechanisms behind men’s sexual decision-making by applying transcranial direct current stimulation (tDCS) to the prefrontal cortex (PFC). Using a sample of 102 heterosexual men, the authors employed a series of first-person vignettes to simulate dating encounters where a woman responded to sexual advances with either a clear refusal or an ambiguous signal. The key contribution is the finding that active tDCS, targeted at the left dorsolateral prefrontal cortex (DLPFC), interacted with these situational cues. Specifically, active stimulation increased reported negative affect and moderated sexual decision-making when participants were faced with explicit refusals. These results suggest that while neuromodulation did not change the initial appraisal of consent, it significantly altered how men emotionally and behaviorally processed social rejection.
The manuscript addresses a highly relevant social issue using a social psychological experimental approach. Integrating brain stimulation with behavioral vignettes allows for a mechanistic exploration of sexual aggression that is often missing from purely observational research. However, the behavioral effects observed in this study are relatively small and do not maintain significance under strict statistical correction. I recommend a revision to ensure the authors address these statistical limitations and provide a more nuanced interpretation of the neuroanatomical targets.
Main issues:
1. In the last paragraph of the Results section, the authors report a significant tDCS interaction but acknowledge it did not survive the Bonferroni correction for multiple comparisons. The authors should focus more of their primary interpretation on the more robust Negative Affect result. Additionally, the authors should either discuss these results in the Discussion section or provide additional robust tests to support the behavioral claims.
2. In the Discussion section, the authors attribute the observed effects specifically to the left DLPFC. However, the tDCS Procedure section describes a 5cm x 5cm bipolar montage (F3/F4) which produces a very diffuse electric field. The authors should revise the Discussion section to acknowledge that the stimulation likely affected a much broader prefrontal network and explain how the rest of the PFC might contribute to or confound the results.
3. The Participants and Procedures section indicates a wide range in the time between the baseline online survey and the in-person visit (2 to 66 days). Although the authors state this was unrelated to outcomes, the nature of the tDCS technique is highly state-dependent. Therefore, tDCS effects are often influenced by the baseline cognitive state of the participant. The authors should provide a supplemental analysis where “days between visits” is included as a covariate in the GLMMs to formally rule out any influence of this variance on the state-dependent effects of tDCS.
4. As noted in the Results, 12 participants received stimulation below the targeted 2mA due to discomfort. A sensitivity analysis comparing the 2mA vs. <2mA subgroups should be reported to ensure that dose-dependency did not skew the results.
Minor Issues:
1. The sample is majority university students and primarily White dominant, which limits the generalizability of the findings to other demographics. Please discuss this limitation.
2. Five individuals did not tolerate the stimulation. Providing a brief table of reported side effects (e.g., itching, tingling) would improve the transparency of the tDCS procedure.
3. The authors note one participant identified as “queer” at follow-up. While sensitivity analyses were done, and it seems that one participant did not alter the result, I would simply exclude this participant from all analysis due to the heterosexual male focus of the study.
4. While the vignettes specified alcohol use as a factor influencing consent ratings, the predicted interaction between alcohol and tDCS was non-significant. The Discussion should address why external PFC stimulation did not interact with alcohol.
5. The Study Outcome section notes that situational elements like alcohol were randomly varied across trials. The authors should clarify if they controlled for the specific order of these vignettes, as the sequence of social cues can influence state-dependent responses to stimulation. 

Decision letter

Thank you for submitting Manuscript ID OXFNSC-2026-006, "An Examination of the Influence of Prefrontal Cortical Brain Stimulation on Sexual Decision Making", to Oxford Open Neuroscience.

Your manuscript has been seen by two reviewers and I have read it myself. As you will see from the reviewer comments below, both reviewers find this work to be of interest and I agree. Therefore, I invite you to respond to the reviewers' comments and revise your manuscript within the next 30 days of today's date.

One issue noted by both reviewers is the variation between participants in stimulation intensity. Please ensure that you address this issue in your revision. The reviewers make several other recommendations, all of which seem reasonable to me, so please consider them carefully.

Please note that this journal operates with transparent peer review. This means that if your submission is accepted for publication, the full peer review history of your article will publish online alongside your article. This includes reviewer comments, editor decision letters, and your author responses.

To revise your manuscript, log into https://mc.manuscriptcentral.com/oxfnsc and enter your Author Centre, where you will find your manuscript title listed under "Manuscripts with Decisions."  Under "Actions," click on "Create a Revision."  Your manuscript number has been appended to denote a revision.

You may also click the below link to start the revision process (or continue the process if you have already started your revision) for your manuscript. If you use the below link you will not be required to login to ScholarOne Manuscripts.

*** PLEASE NOTE: This is a two-step process. After clicking on the link, you will be directed to a webpage to confirm. ***

https://mc.manuscriptcentral.com/oxfnsc?URL_MASK=384e572534eb4c4087d7c0f0941647e5


You will be unable to make your revisions on the originally submitted version of the manuscript.  Instead, revise your manuscript using a word processing program and save it on your computer.  Please also highlight the changes to your manuscript within the document by using the track changes mode in MS Word or by using bold or colored text.

Once the revised manuscript is prepared, you can upload it and submit it through your Author Centre.

When submitting your revised manuscript, you will be able to respond to the comments made by the reviewer(s) in the space provided.  You can use this space to document any changes you make to the original manuscript.  In order to expedite the processing of the revised manuscript, please be as specific as possible in your response to the reviewer(s).

IMPORTANT:  Your original files are available to you when you upload your revised manuscript.  Please delete any redundant files before completing the submission.

Because we are trying to facilitate timely publication of manuscripts submitted to the Oxford Open Neuroscience, your revised manuscript should be uploaded within the next 30 days from todays date.  If it is not possible for you to submit your revision in a reasonable amount of time, we may have to consider your paper as a new submission.

Once again, thank you for submitting your manuscript to Oxford Open Neuroscience and I look forward to receiving your revision.

Authors’ Response

Dear Editors and Reviewers,

We would like to first thank the Editor and Reviewers for their thorough review of our work as well as their helpful comments. We have carefully revised our manuscript and supplementary materials to be attentive to the questions and issues raised in the review. Considerable attention and revision have been directed at addressing questions regarding the differing level of stimulation intensity that some individuals received. This work now includes more description in the Methods section for why this arrangement occurred, additional sensitivity analyses (See Supplementary Tables 11-14) that demonstrate that the pattern of statistical effects endure when excluding individuals who did not receive 2.0 mA stimulation in the active condition, and finally we explicitly review limitations associated with these stimulation procedures in the discussion. Other notable changes to the manuscript include revision of our interpretations regarding the impact of stimulation on specific cortical sites in the prefrontal cortex, a reduced focus in the discussion of stimulation effects on the decision making outcome, limitations associated with this sample and issues of generalizability, as well as multiple other adjustments to language, study description, and statistics reporting (e.g., we now include a stimulation side effects table) based on the feedback of the Reviewers. All changes made to the manuscript have been tracked to enable efficient inspection of the revision. Furthermore, the responses outlined below include page numbers where any changes made can be found in the tracked manuscript.

Below we list each individual comment made by the Editor or Reviewers alongside our responses. Finally, we thank Oxford Open Neuroscience for the continued interest in and consideration of our manuscript for publication. Please let us know if any other concerns arise during the review process. We thank you all again for your time and efforts.

Editor Comment:

• COMMENT: One issue noted by both reviewers is the variation between participants in stimulation intensity. Please ensure that you address this issue in your revision.

RESPONSE: We have made detailed changes to address the issues raised by both Reviewers in the manuscript. First, we have given more detail about the process by which some individuals received lower levels of stimulation intensity. Specifically, some participants requested lower levels of stimulation for their comfort which was possible per our experimental protocol. Next, we conducted sensitivity analyses where the individuals who received less than 2.0mA stimulation in the active group were excluded. These results are given in Supplementary Tables 11-14 and are referred to in the body of the manuscript. Finally, we now raise this issue of stimulation intensity as a limitation in the discussion (See p. 24), along with other methodological issues pertaining to this study’s tDCS methodology.

Reviewer 1
• COMMENT: The abstract contains most of the necessary details, please add a few words onto, or change the wording at the end of the sentence that starts “Active stimulation significantly….” so that the novice reader can understand the direction of effect e.g., reduced negative affect after stimulation/neuromodulation.

RESPONSE: We would like to thank Reviewer 1 for their feedback. We have modified the first sentence of the abstract based on their comment, with an emphasis on describing the directionality of the interaction effect: "Neuromodulation interacted with the communication response type of the woman in the vignette (e.g., ambiguous vs. refusal) to influence participants' reports of negative affect and sexual decision making. For example, participants that received a refusal from the woman while receiving tDCS were more likely to report greater negative affect and a reduced likelihood of sexual behavior as compared with vignettes including an ambiguous response."

• COMMENT: Page 5 line 38: The author mentions “downstream social behaviour” for the first time. For clarity please explain this phrase/term either earlier or briefly in/near that sentence. This is done in part in the next sentence but need further clarity and/or an example.

RESPONSE: We agree with the Reviewer that the use of “downstream” is ultimately ambiguous and, further, unnecessary in text. The term was originally selected in efforts to describe how cortical functioning informs behavior in social scenarios. We have decided to remove the term “downstream” throughout the manuscript. We now use the next sentence on p. 5 to give examples from Choi et al. (2016) of how tDCS impacts different processes related to social behavior.

• COMMENT: Typo: “Procedures” should be “Procedure”

RESPONSE: Thank you, this has been corrected.

• COMMENT: Page 6 line 6: Explain what you mean by “community members” as this can be interpreted to mean or specific closed community, a wider community or the general public. Perhaps a different terms such as “Members of the public” would be more suitable here.

RESPONSE: This change has been made to the manuscript as described by the Reviewer (See first sentence of p. 6).

• COMMENT: Page 6 from line 15: A good level of eligibility criteria are mentioned. These are typical/conventional of neuromodulation experiments - the novice reader might not know this. It could improve your writing if you say that these are the convention for neuromodulation.

RESPONSE: We agree and we have revised the manuscript to indicate that these eligibility criteria are typically used in the neuromodulation field (See p. 6): “Aside from the requirement that participants be fluent in the English language, other eligibility criteria that are typically used in the neuromodulation field were specified, including…”

• COMMENTS: Page 7 line 15: Explain what you mean by “did not tolerate the stimulation procedures” it is likely that you mean minor itchiness or slight skin irritation/sensitivity. Page 8 line 11: The authors mention that some participants received 2mA, some 1.5mA and some 1mA - please explain why this is the case. This is a big limitation for the interpretation of results. e.g., a 2mA intensity could produce very different results to 1mA.
The authors clearly mention 2mA intensity. Above in the previous section 1.5mA and 1mA are also mentioned. If 2mA was used then the earlier section needs correcting. If 2mA was used (as mentioned in the tDCS procedure) then the other intensities need to be mentioned here too with a clear justification for why there were 3 intensity levels.

RESPONSE: We thank the Reviewer for their attention to and comments on this issue. We have made a series of changes based on their feedback and that of Reviewer 2 that are summarized here. At the outset of stimulation procedures, we conducted a “tolerability test” to confirm that participants could tolerate the stimulation procedures. Individuals who found 2.0mA stimulation to be painful or especially irritating or itchy were given the opportunity to complete stimulation at lower intensities (i.e., 1.5 or 1.0 mA) or not to receive stimulation at all. This led to some individuals not receiving stimulation at the 2.0 intensity and some individuals not receiving stimulation at all. We agree that this is a limitation and we have moved to describe it as such in the discussion. In addition, we have provided sensitivity analyses where we have compared only individuals who received 2.0 mA stimulation to the sham group (which demonstrate that the tDCS interaction effects on the negative affect and decision making outcomes are stable).

With respect to the above comments, we have made the following changes to the Methods section: 1) We now describe all issues related to tolerating the stimulation procedures in a single paragraph on p. 8; 2) we have added more description of how participants opted to receive lower stimulation intensities or no stimulation in that paragraph; 3) and we have also created and provided Supplementary Table 15 based on these comments, which list the reported side effects of stimulation as well as their average intensities (on a scale of 1-10) and durations (in minutes). We hope that these changes clarify these questions and concerns raised by the Reviewer.

• COMMENT: Page 8 line 13: The idea about asking participants which type of stimulation/sham they received was a nice idea. The authors needs to explain whether the participants were novices or not to neuromodulation - if they were then they would not understand the relevant terms.

RESPONSE: We have clarified in text that participants were informed that there was a chance that they would not receive active stimulation during the consent process (See p. 8). We did not ask participants whether they were naïve to stimulation procedures and cannot report on this issue. However, this tDCS study was the first of its kind at our institution, perhaps suggesting that the population of young men in this study were unlikely to have received brain stimulation procedures elsewhere.

• COMMENT: It sounds as if ‘offline stimulation’ was used. Please justify why this was used compared to ‘online stimulation’ (i.e., task during stimulation). There is plenty of literature that can be used for this.

RESPONSE: We would argue that the effects observed here are more likely to constitute online stimulation. Participants were first asked to receive 10 minutes of stimulation without completing any task (which could be called offline stimulation). Immediately after these first ten minutes, participants continued to receive stimulation while completing the vignette task (receiving approximately 20 minutes of online stimulation). We believe our results are indicative of an online effect, especially since tDCS condition and task vignette details interacted together to predict negative affect. However, we cannot experimentally disentangle between the offline and online phases of stimulation and their effects on the outcomes in this study. Therefore, we have moved to do the following in response: 1) We have described the stimulation procedure as consisting both of offline and online simulation phases (See p. 11). 2) We now describe this as a limitation alongside the stimulation intensity issues raised previously in a new paragraph in the discussion pertaining to limitations regarding our procedures involving tDCS (See p. 24).

• COMMENT: No ramp-up/down period is mentioned. If this was used please mention it, if not say it was not used.

RESPONSE: A ramp-up/ramp-down procedure was used and is now described in the tDCS Procedure section on p. 11: “A stimulation intensity of 2 mA was targeted in the current work and a 30-second ramp-up/ramp-down procedure was used in both the sham and active procedures.”

• COMMENT: Add the duration of the task. This is important due to the duration of the effect/influence of offline stimulation.

RESPONSE: The task was not fixed in length and proceeded for as long as participants needed to complete it. It required approximately 15-20 minutes for participants to complete the task. The average duration of time taken to complete the task has been added to the manuscript (See pp. 11-12). Additionally, the variable duration of the task is also now described as an experimental limitation that affects disambiguation of offline/online effects (See pp. 24-25). We also note that comparing the effect of offline/online stimulation could be fertile grounds for future investigation in this area.

• COMMENT: The discussion section is generally well written although the following could be considered to improve… The issue mentioned above around the different current intensities needs to be addressed as a limitation of this work.
RESPONSE: We agree with the Reviewer and based on their comments, as well as those of Reviewer 2’s, we have restructured the discussion and included two additional paragraphs of limitations regarding this study’s tDCS methodology as well as limitations pertaining to the study sample (See pp. 24-26 for associated changes).

Reviewer 2

• COMMENT: In the last paragraph of the Results section, the authors report a significant tDCS interaction but acknowledge it did not survive the Bonferroni correction for multiple comparisons. The authors should focus more of their primary interpretation on the more robust Negative Affect result. Additionally, the authors should either discuss these results in the Discussion section or provide additional robust tests to support the behavioral claims.

REVISION: We thank the Reviewer for their attention to this matter and we have endeavored to revise much of the discussion to focus first on negative affect and to further indicate that the behavioral claims are speculative as the finding did not survive Bonferroni correction.

• COMMENT: In the Discussion section, the authors attribute the observed effects specifically to the left DLPFC. However, the tDCS Procedure section describes a 5cm x 5cm bipolar montage (F3/F4) which produces a very diffuse electric field. The authors should revise the Discussion section to acknowledge that the stimulation likely affected a much broader prefrontal network and explain how the rest of the PFC might contribute to or confound the results.

REVISION: We appreciate the Reviewer’s comments on this matter and we have made several changes in the discussion in response. 1) We have noted that our interpretations regarding the DLFPC are speculative and require additional testing in the second paragraph (See pp. 17-18). 2) We have reordered the subsequent paragraphs such that we immediately address issues pertaining to the diffuse electric field and network-level activity following speculation regarding the DLPFC (See tracked changes on pp. 17-20). 3) We ultimately conclude that we modulated the PFC, in general, rather than a specific cortical substrate and we encourage future investigation that can clarify the contribution of specific anatomical regions to the pipeline of cognitive-affective processing of sexual scenarios (See pp. 18-19). 4) We further raise the diffuse electric field used here as a limitation towards the close of the manuscript (See p. 24).

• COMMENT: The Participants and Procedures section indicates a wide range in the time between the baseline online survey and the in-person visit (2 to 66 days). Although the authors state this was unrelated to outcomes, the nature of the tDCS technique is highly state-dependent. Therefore, tDCS effects are often influenced by the baseline cognitive state of the participant. The authors should provide a supplemental analysis where “days between visits” is included as a covariate in the GLMMs to formally rule out any influence of this variance on the state-dependent effects of tDCS.

REVISION: We thank the Reviewer for this comment. We had previously tested days between study visits as a covariate in GLM models and found it to be unrelated but did not specifically report that we tested it as such in the manuscript. In response to this comment, however, we have opted to include Supplementary Table 10, which lists the nonsignificant fixed effects results for the Days between Visits variable in the final Consent, Positive Affect, Negative Affect, and Decision Making models. We also note in Supplementary Table 10, as well as here, that including this variable did not change the results or interpretation of other fixed effects values. We have also updated a footnote on p. 7 in the manuscript that the time between visits was found to be unrelated based on the results of additional GLMMs: “1 Time between study visits was found to be unrelated to study outcomes based on the results of additional GLM models that included time as a covariate (See Supplementary Table 10).”

• COMMENT: As noted in the Results, 12 participants received stimulation below the targeted 2mA due to discomfort. A sensitivity analysis comparing the 2mA vs. <2mA subgroups should be reported to ensure that dose-dependency did not skew the results.

REVISION: Consistent with our changes in response to Reviewer 1's concerns, we have provided greater detail as to why some participants received lower doses of tDCS in the methods (e.g., out of comfort) on p. 8. Next, we have added four Supplementary Tables (Supplementary Tables 11-14) reporting GLMM fixed effects while excluding any individuals who received 1.0 or 1.5mA stimulation in the active group. The pattern of effects on the negative affect and decision making outcomes was retained. Finally, we have indicated this issue, as well as others related to stimulation, as a limitation of our work in the discussion (See p. 24).

• COMMENT: The sample is majority university students and primarily White dominant, which limits the generalizability of the findings to other demographics. Please discuss this limitation.

REVISION: We thank the Reviewer for their comment on this issue as we had initially wanted to discuss this sample at greater length but decided not to out of concerns of manuscript length. The limitation regarding the sample’s demographics is now outlined in an additional paragraph in the discussion, and we importantly note that the effects observed in this work may not generalize to high profile contexts of sexual aggression (e.g., forensic sexual offending and intimate partner violence). We have also taken this opportunity to address unique aspects of conducting research with this population of young men at this time, which we believe readers will find interesting (See pp. 25-26).

• COMMENT: Five individuals did not tolerate the stimulation. Providing a brief table of reported side effects (e.g., itching, tingling) would improve the transparency of the tDCS procedure.

REVISION: Based on this feedback and that of Reviewer 1, we have made considerable changes to reporting the tolerability of stimulation procedures (See p. 8). We have also included a table of stimulation side effects in the supplementary materials (See Supplementary Table 15).

• COMMENT: The authors note one participant identified as “queer” at follow-up. While sensitivity analyses were done, and it seems that one participant did not alter the result, I would simply exclude this participant from all analysis due to the heterosexual male focus of the study.

RESPONSE: As the Reviewer notes in their comment, sensitivity analyses did not demonstrate any substantive differences with this individual included vs. excluded. Notably, we also asked participants whether they had any history of sexual intercourse with a man, and this individual did not report any such history - we note this information in the manuscript now on p. 7: “One participant changed their reported sexual orientation from heterosexual at screening to queer at follow-up, however this individual reported no prior history of sexual intercourse with a man.”

While we could exclude the individual, we think it is worthwhile to include them in the analyzed sample because this man could feasibly find himself in a heteronormative sexual encounter. We are inclined to leave the individual in this analyzed sample for the purposes of this study, as understanding the decision making of men who find themselves in heteronormative sexual encounters is of critical public health interest to prevent dating violence.

Finally, more conceptually, sexual orientation is a complex construct. This individual initially identified as heterosexual and later as queer. It is possible that he holds a primarily heterosexual interest but also holds at least some attraction to individuals of the same sex, which may also be true of other participants recruited in this sample. All in all, we argue to include this individual for the purposes of this study.

• COMMENT: While the vignettes specified alcohol use as a factor influencing consent ratings, the predicted interaction between alcohol and tDCS was non-significant. The Discussion should address why external PFC stimulation did not interact with alcohol.

RESPONSE: We thank the Reviewer for this comment as well and we have revised our discussion of the tDCS interaction to address this point. We make the argument that women's communication may have held a heightened cognitive relevance to men's responding based on sexual script theory. We now contrast the null findings regarding alcohol use and intimacy attained with these points, suggesting that these contextual details may be less salient on men’s cognitive processing generally and therefore have a lesser opportunity for tDCS to interact with than factors that were clearly pertinent (See p. 21): “Lastly, stimulation interacted with women’s responses and it is unclear what kind of simultaneous cognitive effects that this may have had on men. It may be that stimulation caused men to attend even more closely towards factors that they believed to be stereotypically associated with or salient to their sexual goals. Under such a scenario, men’s awareness of overt situational cues such as a woman’s refusal (or lack thereof) may have been enhanced by tDCS. In contrast, other contextual details that appeared relevant to the sample’s consent appraisals (i.e., the level of alcohol use or intimacy attained in the scenario) may have lacked sufficient cognitive salience to interact with tDCS to direct their negative affective reactions or decision making. Alternatively, stimulation may have simply changed the intensity of men’s behavioral and negative affective responding in provocative scenarios such as a sexual refusal. Future study that can investigate how PFC manipulations affect the processing of sexual information in the moment may clarify the cognitive-affective determinants of aggressive decision making.”

• COMMENT: The Study Outcome section notes that situational elements like alcohol were randomly varied across trials. The authors should clarify if they controlled for the specific order of these vignettes, as the sequence of social cues can influence state-dependent responses to stimulation.

RESPONSE: Thank you for this comment – we have moved to indicate that the order in which vignette information was presented was fixed in this study and that it is unclear how this might influence study outcomes. We note this as a limitation related to our vignette paradigm on p. 25 (See below). Notably, we also reference a work from Willis & Jozkowski (2022) that tackles the issue of how the sequential presentation of information in factorial vignettes can impact sexual consent appraisals: “Additionally, this study presented situational elements in vignettes in a fixed order, where a night out gradually proceeds before an ambiguous sexual encounter begins. Other research has demonstrated that the order in which situational elements are presented affects appraisals of sexual consent (Willis & Jozkowski, 2022) and it is unclear how the preset order of situational information in these vignettes may have influenced study outcomes.”


Round 2 reviews
Reviewer 2
Recommendation – Accept
Comments to author:
I thank the authors for a careful and thorough revision. The revised manuscript addresses the principal concerns raised in the first round of review. The Discussion has been restructured to foreground the Negative Affect result and to explicitly caveat the decision-making finding as failing to survive Bonferroni correction. The neuroanatomical interpretation has been meaningfully revised: the paragraph that over-attributed the observed effects to the left DLPFC has been removed, and a new passage acknowledging that the bilateral electrode montage produced a diffuse electric field likely engaging a broader prefrontal network has been added in its place, supported by an additional citation. Supplementary analyses formally ruling out the influence of the interval between study visits and of stimulation dose have been provided, and the limitations section has been expanded to address sample generalizability and vignette ordering. A side effects table has also been added.
I recommend acceptance subject to minor edits. The two remaining concerns below are editorial in nature and do not require new analyses.
1. In the revised Results section, the decision-making outcome is newly described as “the likelihood of aggressive behavior.” This phrasing is stronger than what the vignette items appear to directly measure, which assess the likelihood of continuing sexual advances rather than aggressive behavior per se. The authors should either briefly justify this relabeling with reference to the item wording, or revert to the more conservative phrasing used in the original submission.
2. Footnote 1 states that the interval between study visits was “unrelated to study outcomes based on the results of additional GLM models that included time as a covariate (See Supplementary Table 10).” Reporting the relevant test statistics in the footnote itself, in addition to the supplementary table reference, would allow readers to evaluate this claim without consulting the supplementary materials. 
Decision letter
Thank you for your revised manuscript OXFNSC-2026-006.R1 entitled "An Examination of the Influence of Prefrontal Cortical Brain Stimulation on Sexual Decision Making", which you submitted to the Oxford Open Neuroscience.

Your manuscript was re-reviewed by one of the original reviewers, who found the revision very thorough. They have recommended acceptance, subject to two minor edits. I am therefore issuing a 'minor revision' decision so that you have the opportunity to respond to these points. Once I receive your revised manuscript I will endeavour to send a decision quickly, without further review. Please submit your revision within the next 30 days, or contact the editorial office if you require an extension.

Please note that this journal operates with transparent peer review. This means that if your submission is accepted for publication, the full peer review history of your article will publish online alongside your article. This includes reviewer comments, editor decision letters, and your author responses.

To revise your manuscript, log into https://mc.manuscriptcentral.com/oxfnsc and enter your Author Centre, where you will find your manuscript title listed under "Manuscripts with Decisions."  Under "Actions," click on "Create a Revision."  Your manuscript number has been appended to denote a revision.

You may also click the below link to start the revision process (or continue the process if you have already started your revision) for your manuscript. If you use the below link you will not be required to login to ScholarOne Manuscripts.

*** PLEASE NOTE: This is a two-step process. After clicking on the link, you will be directed to a webpage to confirm. ***

https://mc.manuscriptcentral.com/oxfnsc?URL_MASK=a78cb93293b545c394b18fc1c9bd0908

You will be unable to make your revisions on the originally submitted version of the manuscript.  Instead, revise your manuscript using a word processing program and save it on your computer.  Please also highlight the changes to your manuscript within the document by using the track changes mode in MS Word or by using bold or colored text.

REMINDER: if you have not already done so, when submitting your revised manuscript, please include a short alternative-text description under the figure legend for each figure. Alt-text is required to ensure the accessibility of our publications. Guidance is available on the journal’s Author Guidelines webpage and at https://academic.oup.com/pages/for-authors/journals/preparing-and-submitting-your-manuscript

Once the revised manuscript is prepared, you can upload it and submit it through your Author Centre.

When submitting your revised manuscript, you will be able to respond to the comments made by the reviewer(s) in the space provided.  You can use this space to document any changes you make to the original manuscript.  In order to expedite the processing of the revised manuscript, please be as specific as possible in your response to the reviewer(s).

IMPORTANT:  Your original files are available to you when you upload your revised manuscript.  Please delete any redundant files before completing the submission.

Because we are trying to facilitate timely publication of manuscripts submitted to the Oxford Open Neuroscience, your revised manuscript should be uploaded within the next 30 days from todays date.  If it is not possible for you to submit your revision in a reasonable amount of time, we may have to consider your paper as a new submission.

Once again, thank you for submitting your manuscript to Oxford Open Neuroscience and I look forward to receiving your revision.


Authors’ response
We once again thank the Editor and Reviewer for their attention and feedback throughout this submission process. We have addressed the two remaining concerns identified by the Reviewer in the manuscript and included summary of the changes below:

1. We have opted to revert our language to be more conservative in the results section and to describe the decision making outcome as "participants' hypothetical behavior" instead of "the likelihood of aggressive behavior."
2. We agree with the Reviewer's second comment and we now note that the time between study visits covariate was not significantly associated with outcomes and provide associated test statistics towards this point:
"Time between study visits was not found to be significantly associated with study outcomes (p’s > 0.05) based on the results of additional GLM models that included time as a covariate (See Supplementary Table 10)."

In the currently submitted manuscript, all changes from the first revision have been accepted and these two minor edits have been tracked. Please let us know if we can provide any further clarification on the manuscript and thank you for your attention again.

Round 3 reviews
n/a
Decision Letter:
It is a pleasure to accept your revised manuscript entitled "An Examination of the Influence of Prefrontal Cortical Brain Stimulation on Sexual Decision Making" in its current form for publication in Oxford Open Neuroscience. 

Please note that this journal operates with transparent peer review. This means that  the full peer review history of your article will publish online alongside your article. This includes reviewer comments, editor decision letters, and your author responses.

Next steps
You will receive an email from no-reply@scipris.com within roughly one week. This is your invitation to sign up for an account with SciPris, Oxford University Press’ author portal hosted by Aptara. You will need to create an account if you do not already hold one. Please register or log into your account and follow the online instructions which will guide you through signing your licence and paying the APC. The email and the portal have clearly signposted support options if you need any help during this process.

Please note that SciPris is a completely different system from ScholarOne, so your credentials to submit your manuscript here will not work there. Once you’ve created a SciPris account, you will be able to use it whenever you publish with Oxford Open Neuroscience or any OUP journal. Please note that OUP will only ever request payment for applicable fees be made via SciPris or to an OUP bank account. If you ever have concerns about the legitimacy of a request, please do not hesitate to contact a customer services agent via the SciPris portal or directly via oupsupport@scipris.com.

Thank you for your fine contribution.  On behalf of the Editors of the Oxford Open Neuroscience, we look forward to your continued contributions to the Journal.
